# Aggregation-induced emission of matrix-free graphene quantum dots via selective edge functionalization of rotor molecules

**DOI:** 10.1126/sciadv.ade2585

**Published:** 2023-02-17

**Authors:** Sukki Lee, Jinho Lee, Seokwoo Jeon

**Affiliations:** ^1^ Department of Materials Science and Engineering, KAIST Institute for the Nanocentury (KINC), Korea Advanced Institute of Science and Technology (KAIST), Daejeon 34141, Republic of Korea.; ^2^ Department of Materials Science and Engineering, Korea University, Seoul 02841, Republic of Korea.

## Abstract

Graphene quantum dots (GQDs) are nanosized graphene derivatives with unique photoluminescence (PL) properties that have advantages in optoelectronic applications due to their stable blue light emission. However, aggregation-caused quenching (ACQ) of GQDs limits the practical applications on light-emitting diodes. Here, we suppress the ACQ phenomena of GQDs by reducing the size and converting GQDs into aggregation-induced emission (AIE)–active materials. As the size of GQDs is reduced from 5 to 1 nm, their solid-state PL quantum yields (PLQYs) are improved from 0.5 to 2.5%, preventing ACQ. Two different rotor molecules, benzylamine (BA) and 4,4′-(1,2-diphenylethene-1,2-diyl)diphenol (TPE-DOH), are selectively functionalized by substituting carboxylic acid and carbonyl functional groups. All functionalized GQDs show AIE behaviors with significantly enhanced solid-state PLQYs, up to 16.8%. Afterglow measurements and theoretical calculations reveal that selective functionalization hinders inter- and intramolecular charge transfer, which enhances the fluorescence rate of GQDs and corresponding PLQY.

## Teaser

Graphene quantum dots can be converted into highly luminescent solid-state materials toward blue LEDs.

## INTRODUCTION

Graphene quantum dots (GQDs) have been developed as next-generation candidates for optoelectronics, taking advantage of their biocompatibility (*1*–*3*), thermal and photostability (*2*, *4*), and resistance to oxygen and water (*5*). Most notably, GQDs are known to emit stable blue light (*6*–*9*) through a large bandgap with a nanosized sp^2^-conjugated structure. One of the main challenges of GQDs in realizing effective solid-state lighting is to improve the low photoluminescence (PL) quantum yield (QY). The oxygen functional groups of GQDs can activate multiple charge transfer (CT) states (*10*) that can behave as nonradiative recombination centers, resulting in a low PLQY. In the solid state, reabsorption or energy transfer between adjacent GQDs lowers the PLQY even more significantly (*11*). Therefore, proper modification of GQDs is necessary to not only achieve sufficient charge transport characteristics but also suppress the abovementioned issues arising from GQDs in the solid states to realize high quantum efficiency optoelectronics, such as light-emitting diodes (LEDs).

Typical GQDs in previous literature included carbon-based quantum dots and graphene oxide quantum dots (*2*), which inevitably experience oxidation during synthesis, resulting in random formation of sp^2^ domains and uncontrolled functional groups. It is difficult to tune the PL properties of such quantum dots due to the complex PL origins and various CT states generated by uncontrollable molecular configurations, which limits their practical use in LEDs. Moreover, our group previously reported GQDs fabricated from graphite intercalation compounds (GICs) (*12*–*17*) that have a highly preserved sp^2^ domain with controlled oxidative functional groups (*4*, *10*, *18*–*20*). On the basis of their characteristic structures, blue emission can be achieved regardless of their physical size with a settled PL origin (*9*); this structure is called a subdomain (*18*). In addition, precisely controlled oxidative functional groups allow the regulation of CT states, which effectively promotes triplet-mediated emission, including room-temperature phosphorescence (RTP) and thermally assisted delayed fluorescence (TADF) (*19*). With these findings, the realization of the first GQD-based LED and alternating-current powder electroluminescent (ACPEL) device by embedding GQDs into an adequate matrix material such as poly(9-vinylcarbazole) (*10*) and boron oxynitride (*20*) was possible. Nevertheless, most matrices that stabilize the emission of GQDs have strong dielectric properties (*21*–*23*) and can cause undesirable interactions with GQDs, such as exciplexes (*24*) and CT complexes, which impedes the distinctive emission of GQDs. Therefore, it is critical to retain the luminescence properties of GQDs in the solid state similar to their solution counterparts without introducing any matrices (*4*). Realization of stable solid-state emission of GQDs, however, has rarely been reported due to the strong π-π stacking nature of graphene sp^2^ structures, which provokes severe aggregation-caused quenching (ACQ) (*11*). To improve the solid-state PLQY even more than that in solution, it is essential to rigorously manage not only steric hindrance (*4*) but also CT states (*25*).

Since their discovery by B. Z. Tang and colleagues in 2001 (*26*), aggregation-induced emission (AIE) phenomena have emerged as a breakthrough to resolve ACQ in organic materials (*27*), making their application in LEDs intriguing. Unlike conventional π-conjugated molecules, AIE-active materials in the solid state prefer specific configurations that maximize steric hindrance through mechanisms such as restriction of intramolecular rotation (RIR) (*28*) or restriction of intramolecular vibration. Recently, AIE combined with triplet-mediated luminescence mechanisms such as RTP (*29*–*31*) and TADF (*32*–*34*) was highlighted as a promising alternative to heavy-metal–based phosphorescent materials with effective control over triplet manifolds. These tendencies accelerate the development toward the ultimate goal of organic LEDs with exciton utilization as high as approximately 100% without any host materials (*32*, *35*). Nevertheless, designing AIE molecules requires complicated reaction schemes and careful consideration of their molecular configurations, such as donor-π-acceptor–type designs (*33*) and perarylations (*36*).

Inspired by synthetic techniques used to design AIE materials, we propose simple synthesis strategies to convert GQDs from ACQ to AIE-active materials using various rotor molecules. Two strategies are chosen to realize AIE-active GQDs: (i) size reduction to suppress π-π stacking and (ii) selective edge functionalization using rotor molecules. As the size of the GQDs is reduced from 5 to 1 nm (pristine GQDs), ACQ is suppressed to obtain stable matrix-free fluorescence with an RTP afterglow. Using these pristine GQDs, two selective edge functionalizations, (i) carboxylic acid groups into amide or ester groups and (ii) carbonyl groups into amine or ether groups, enable the attachment of rotor molecules such as benzylamine (BA) and 4,4′-(1,2-diphenylethene-1,2-diyl)diphenol (TPE-DOH). Rotor-functionalized GQDs have intriguing AIE properties in powder forms, in sharp contrast to the ACQ properties of nonfunctionalized GQDs. In particular, density functional theory (DFT) calculations reveal that substitutions of carbonyl groups into those rotor molecules suppress intramolecular CT, reducing intersystem crossing (ISC). The tunable ISC rate helps realize GQDs with variable lifetimes and high PLQYs originating from the combined effects of RTP, TADF, and intrinsic fluorescence. Blue GQDs (~1 nm) in powder form, without any matrices introduced, maintain a high PLQY of up to 16.8%, representing the expanding the possibility of using blue GQDs for solid-state lighting in the near future.

## RESULTS

### Experimental concepts for AIE-active GQDs

**Fig. 1.**
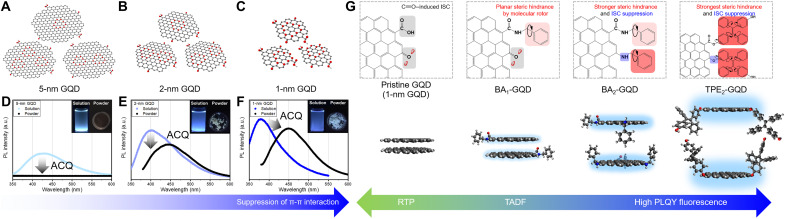
Strategies to synthesize AIE-active GQDs via size control and selective functionalization. Configurations and PL spectra (λ_ex_ = 325 nm) of (**A** and **D**) 5-nm GQDs, (**B** and **E**) 2-nm GQDs, and (**C** and **F**) 1-nm GQDs. The insets show real images of the corresponding GQDs in solution and powder under irradiation with a 365-nm ultraviolet (UV) lamp. a.u., arbitrary units. (**G**) Schematic illustrations of the selective functionalization of GQDs with rotor molecules. The top parts and the bottom parts of the images represent the configuration and packing structure of each GQD, respectively. The key features of each GQD are emphasized with boxes of different colors and comments (gray, ISC promoter; red, steric hindrance; blue, ISC suppressor).

Figure 1 illustrates strategies to realize AIE-active GQDs; these strategies included size control (Fig. 1, A to F) and selective edge functionalization of GQDs with various rotor molecules (Fig. 1G and fig. S1). All GQDs presented hereafter were synthesized from GICs using potassium sodium tartrate (KNaC_4_H_4_O_6_·4H_2_O) as an intercalant (*10*, *18*). In correspondence with our previous studies, these GQDs were used because they can emit stable blue light in the solution stemming from their characteristic structures, as we mentioned in the introduction. Because the π-π interaction of GQDs results from the strong intermolecular overlap between π-electrons in the sp^2^ carbon structures of each GQD (*4*, *37*), we reduced the size of the GQDs from 5 to 1 nm to suppress π-π interactions by decreasing π conjugation (Fig. 1, A to C). After the GQDs less than 10,000 Da (< 5 nm) were filtered with a centrifugal filter (Amicon Ultra15 Centrifugal Filters), the GQDs were further separated by sizes of 5, 2, and 1 nm through dialysis with membranes with different molecular weight cutoffs (MWCOs) (further details are provided in Materials and Methods and fig. S1). The sizes of the as-separated GQDs were assessed by transmission electron microscopy (TEM) images with corresponding size distributions and images (fig. S2). The size effect on π-π interactions upon aggregation was confirmed with the PL properties by comparing those of GQDs in solution and powder. As shown in Fig. 1 (D to F) and table S1, the peaks (λ_em, soln_) of the PL spectrum of the GQD solutions are observed at 410, 400, and 388 nm with a decrease in size from 5 to 1 nm, with a slightly increased solution PLQY (Φ_F,soln_), to 6.8%, for pristine GQDs. The blueshift in the PL of the solution can be attributed to the overall decrease in π conjugations as the lateral size of the GQDs decreases. The PL of the solutions exhibits relatively stable blue emission regardless of the GQD size, while the PL behavior of the powders strongly depends on their sizes due to the different overlaps of π-electrons. Figure 1D shows that the 5-nm GQDs in powder show no distinctive peak because of severe quenching, with negligible Φ_F,powder_ of 0.5% (table S1), which is typical ACQ behavior; this is also demonstrated by the nondetectable PL from the powders in a digital image (inset of Fig. 1D). The Φ_F,powder_ values of the 2-nm GQDs and pristine GQDs in powder are 1.2 and 2.5%, which are lower than those of their solution counterparts (Fig. 1, E and F); nevertheless, their λ_em, powder_ values are enhanced by 2.2- and 5.0-fold in comparison to that of the 5-nm GQDs. The PL properties of the powders prove that reducing the size of GQDs can effectively mitigate π-π interactions between GQDs. Surface analysis of the GQDs with different sizes was conducted to investigate how oxidation affects the PL properties. High-resolution x-ray photoelectron spectroscopy (XPS) measurements (fig. S3 and table S2) demonstrate that the oxygen content increases from 26.12 (5-nm GQDs) to 42.34% (pristine GQDs), and additionally, with decreasing size, the sum of C═O and ─COOH group ratio increases from 11.25 to 27.72%. This occurs because the ratio of edge-residing functional groups, such as C═O and ─COOH, increases with decreasing size due to the increase in the edge ratio with respect to the basal plane area of the GQDs. Raman spectroscopy measurements were conducted to verify the influences of edge sites (fig. S4). As the size of the GQDs decreased from 5 to 1 nm, the corresponding D to G raito (*I*_D_/*I*_G_) ratio increased from 0.64 to 0.77. An increased *I*_D_/*I*_G_ ratio reflects the increased edge-residing oxygen functional groups that mitigate E_2g_ mode stretching from sp^2^ structures, which is consistent with the XPS results. It can be noticed that a new peak at 1450 cm^−1^ appears for 1-nm GQDs that cannot be observed from other sizes, which may be due to the splitting of D bands by edge carbonyl groups (*38*) in the GQDs as small as coronene (*39*). Note that, for GIC-based GQDs, even if the oxidation degree or the number of surface groups increases, their blue emission is maintained, implying that the locally excited (LE) states of GIC-based GQDs consist mainly of subdomain structures (*18*).

Schematic illustrations showing the conversion of pristine GQDs to AIE-active materials by selective edge functionalization are provided in Fig. 1G. The top part of Fig. 1G describes consecutive edge functionalizations of pristine GQDs using rotor molecules, such as BA and TPE-DOH. Subscript “1” refers to GQDs that involve the functionalization of pristine GQDs with only carboxylic acid, and subscript “2” refers to GQDs functionalized with both carboxylic acid and carbonyl groups. BA_1_-GQDs were synthesized using carbodiimide cross-linker chemistry (*4*, *40*), attaching BA groups onto pristine GQDs by forming amide bonds. BA_2_-GQDs were synthesized using reductive aminations (*41*) to substitute carbonyl groups into the amine group of BA addtionally from BA_1_-GQDs (fig. S1). To synthesize TPE_2_-GQDs, pristine GQDs were subjected to successive Steglich esterifications (*42*) and reductive etherifications (*43*) with TPE-DOH (fig. S1). Further experimental details are provided in Materials and Methods. The overall objectives for each functionalization are as follows: (i) suppression of intermolecular CT by maximizing steric hindrance and (ii) suppression of ISC by eliminating ISC-promoting functional groups. According to our previous study, the C═O derivatives (Fig. 1G, gray boxes) of GQDs can promote ISC to realize RTP, so we rationalize that, by replacing C═O derivatives with alternative functional groups without n orbitals characteristic perpendicular to π orbitals, intersystem crossing rate (*k*_ISC_) would be reduced, hence increasing the fluorescence QY of GQDs. Moreover, BA (*44*) and TPE-DOH (Fig. 1G, red boxes) (*45*) have previously been reported to increase the internal conversion rate (*k*_IC_) in “good” solvents owing to the free rotation of phenyl groups, which can activate AIE and increase steric hindrance. The BA group only introduces in-plane steric hindrance when substituted by amide bonds in BA_1_-GQDs, in contrast, direct substitution of C═O into the BA group reduces ISC (Fig. 1G, blue boxes) and induces out-of-plane steric hindrance at the same time(Fig. 1G, red boxes), improving the fluorescence QY of BA_2_-GQDs compared with that of BA_1_-GQDs (Fig. 1G). Among the various GQDs presented TPE_2_-GQDs are expected to maximize steric hindrance, with four phenyl groups that can freely rotate; accordingly TPE_2_-GQDs are expected to show the highest QY of fluorescence.

### PL properties of GQDs

**Fig. 2.**
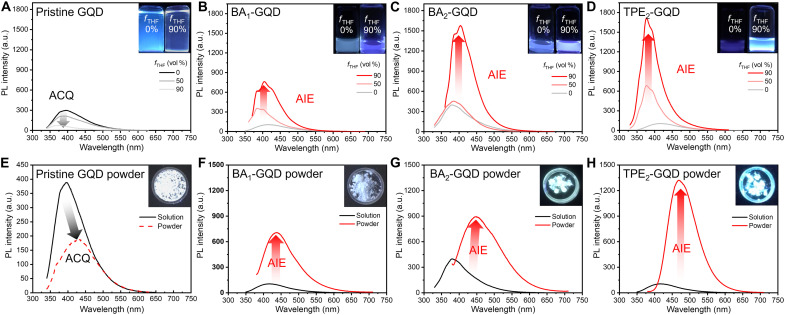
Emission properties of GQDs in various states. Emission spectra of (**A**) pristine GQD, (**B**) BA_1_-GQD, and (**C**) BA_2_-GQD with different volume ratios of THF (*f*_THF_) in water and (**D**) TPE_2_-GQD with different volume ratios of THF (*f*_THF_) in DMSO (inset: photograph of each type of GQD at *f*_THF_ = 0% and *f*_THF_ = 90% under 365-nm excitation). vol %, volume percent. Solid-state emission spectra of (**E**) pristine GQDs, (**F**) BA_1_-GQDs, (**G**) BA_2_-GQDs, and (**H**) TPE_2_-GQDs with respect to their solution counterparts (inset: photograph of GQD powders under 365-nm excitation).

The emission spectra for edge-functionalized GQDs in various aggregated states are provided in Fig. 2. The volume fraction of tetrahydrofuran (THF) (*f*_THF_) in a THF-in-water system was used to control the aggregation degree of pristine GQDs, BA_1_-GQDs, and BA_2_-GQDs because these GQDs are highly hydrophilic, thus the miscible solvent here is water. It should be noted that, because of the functionalization of pristine GQDs with TPE-DOH, the hydrophilicity of TPE_2_-GQDs became lower than that of pristine GQDs. This made TPE_2_-GQDs more miscible in dimethylsulfoxide (DMSO) than water; thus, a THF-in-DMSO system was selected for the TPE_2_-GQDs instead. Greater *f*_THF_ induces greater aggregation, therefore, decreasing the PL intensity of the pristine GQDs as f_THF_ becomes larger (Fig. 2A), likewise the PL of the pristine GQD powder is quenched (Fig. 2E), in accordance with the data shown in Fig. 1F. In notable contrast with the pristine GQDs, BA_1_-GQDs, BA_2_-GQDs, and TPE_2_-GQDs exhibit remarkable increases in PL intensity as *f*_THF_ increases (Fig. 2, B to D), with a similar enhancement of PL in powder (Fig. 2, F to H). The Φ_F,soln_ for BA_1_-GQDs, BA_2_-GQDs, and TPE_2_-GQDs correspond to 3.2, 5.1, and 4.4%, respectively, and these values are even lower than that of pristine GQDs; this observation can be ascribed to the free rotation of BA and TPE groups at the edges promoting *k*_IC_ when the GQDs are dispersed in miscible solvent (table S3). However, the Φ_F,agg_ for BA_1_-GQDs, BA_2_-GQDs, and TPE_2_-GQDs are enhanced to 8.5, 15.6, and 17.2%, respectively, and these values are 2.7-, 3.1-, and 3.9-fold higher than the Φ_F,soln_ of the corresponding GQDs (table S3). Intriguingly, these increases in PLQY in aggregates are accompanied by changes inthe PL spectra structures and their wavelength dependency. As shown in fig. S5, all GQDs showed the following representative behaviors: (i) structureless emission and (ii) excitation-wavelength dependencies of PL peak positions, which are observed in most GQDs due to the CT characteristics (*10*, *46*) or electron-vibration coupling (*47*). On the contrary, for aggregates at *f*_THF_ = 90 (fig. S6), AIE-active GQDs show vibrational peaks concurrent with excitation-wavelength independent behaviors (fig. S6, B to D). These behaviors can be attributed to the emergence of LE states of GQDs as a consequence of reduced electron-vibration coupling through the “RIR” of rotor molecules at the edges (*28*) that effectively suppress the *k*_IC_ as well as suppression of CT states in a relatively nonpolar medium (*48*) by increasing THF ratios in H_2_O or DMSO. This suppression of CT states in aggregates (*f*_THF_ = 90) can also be evaluated by reduction of full width at half maximum (FWHM; Δλ) values in table S3 calculated from spectra in Fig. 2. Δλ is effectively reduced from 101, 87, and 100 nm in their monomer state (*f*_THF_ = 0) to 78, 74, and 58 nm in their aggregated state (*f*_THF_ = 90) for BA_1_-GQDs, BA_2_-GQDs, and TPE_2_-GQDs, respectively (table S3).

Likewise, enhancements in the Φ_F,powder_ versus Φ_F,soln_ are observed in Fig. 2 (F to H) and table S3. The Φ_F,powder_ for BA_1_-GQDs, BA_2_-GQDs, and TPE_2_-GQDs are enhanced to 7.9, 15.6, and 16.8%, respectively, and these values are 3.2-, 6.2-, and 6.7-fold the Φ_F,powder_ of the corresponding GQDs. As can be observed from fig. S7, all GQDs show excitation-dependent behaviors that can also be observed in *f*_THF_ = 0, retaining their characteristic energy structure from pristine GQDs. This indicates that emissions of powder stem from GQDs, not from BA or TPE-DOH groups. The Δλ of GQDs are broader in powder than that in *f*_THF_ = 90, most likely as a result of their medium switching from nonpolar to more polar in powder, but TPE_2_-GQD powder still shows a reduced Δλ of ~92 nm compared to that of pristine GQD powder at ~125 nm (table S3). These results suggested that CT states can also be suppressed even in their strongly aggregated states. The blue emission properties of AIE-active GQDs throughout the different states can also be visualized by Commission International de l’Eclairage coordinates in fig. S8 (A to D), ranging from (0.174, 0.158) for BA_1_-GQDs to (0.167, 0.236) for TPE_2_-GQDs even in their powder form (fig. S8C). The unconventional increase in the PLQY of rotor-functionalized GQDs (BA_1_-GQDs, BA_2_-GQDs, and TPE_2_-GQDs) upon aggregation indicated that pristine GQDs were successfully converted from ACQ to AIE-active material, which was realized with the RIR of each rotor at the edges (*28*). This AIE phenomenon can also be verified with the α_AIE_ value (the calculation detail is given in eq. S1 and the values are given in table S3) (*49*), and the α_AIE_ values were calculated to be 0.36 (pristine GQDs), 2.47 (BA_1_-GQDs), 3.05 (BA_2_-GQDs), and 3.82 (TPE_2_-GQDs), which corresponds with the results from Fig. 2 (E to H). 

### Characterization of selectively functionalized GQDs

**Fig. 3.**
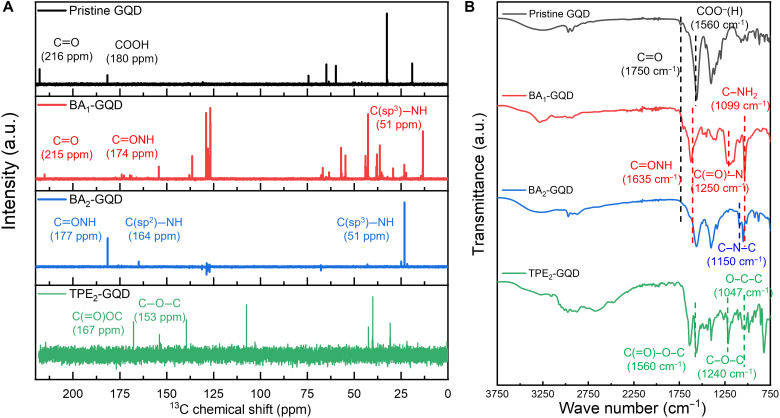
Characterization of various GQDs. (**A**) ^13^C NMR spectra. (**B**) FT-IR spectra of various GQDs.

To corroborate selective edge functionalization, the as-synthesized GQDs were characterized by ^13^C nuclear magnetic resonance (NMR) and Fourier transform infrared (FTIR) spectroscopy. As shown in Fig. 3A, the ^13^C NMR chemical shifts corresponding to the sp^2^ carbon structure [125 parts per million (ppm)] and ─OH group (60 to 70 ppm) remain unchanged for all four GQDs (*50*). Thus, it was confirmed that sp^2^ carbon structures and ─OH groups in pristine GQDs do not undergo any important chemical interactions. Carbonyl (C═O) groups (215 ppm) and carboxylic acid (─COOH) groups (180 ppm) are detected in pristine GQD (*51*). For BA_1_-GQDs, the amide [─C(═O)NH─] group (174 ppm) can be observed with the chemical shift of the carboxylic acid group (*52*), but the carbonyl group was still observed at 210 ppm, successfully proving selective functionalization occurred only with the carboxylic acid group into the amide group. For BA_2_-GQDs, a C═O chemical shift was not present, and a chemical shift from the amine [─C(sp^2^)─NH─] group (160 ppm) can be observed, which indicates full substitution of the C═O group into the amine group. It should be noted that, for BA_1_-GQDs and BA_2_-GQDs, ─C(sp^3^)─NH─ (51 ppm) belongs to the amine group in the BA rotors; thus, BA moieties are successfully functionalized on the surface both on BA_1_-GQDs and BA_2_-GQDs. The ester [─C(═O)OC─] groups (167 ppm) and ether (─C─O─C─) groups (153 ppm) were detected in TPE_2_-GQDs as a result of esterification as well as reductive etherification between pristine GQDs and TPE-DOH. Selective functionalizations of GQDs were also examined by FTIR spectra (Fig. 3B). FTIR peaks corresponding to C═O and ─COOH in pristine GQDs were found at 1750 and 1560 cm^−1^, respectively. The C═O peak was present for BA_1_-GQDs, and the ─COOH peak was replaced with the ─CONH─ peak at 1635 cm^−1^, in accordance with the result from ^13^C NMR. The FTIR spectra of BA_2_-GQDs, on the other hand, exhibited no apparent peaks of C═O at 1750 cm^−1^ but revealed peaks corresponding to the C─N─C functional group at 1150 cm^−1^ in which each carbon belongs to both GQDs and BA moieties. For TPE_2_-GQDs, C(═O)─O─C (1650 cm^−1^) from esterification of the carboxylic acids of pristine GQDs and C─O─C (1240 cm^−1^) from ether groups linking GQDs and TPE-DOH, with no apparent peak for C═O at 1750 cm^−1^, prove the consecutive esterification and reductive etherification of GQDs with TPE-DOH.

To delve into how these functionalizations affect the overall intermolecular distance, small-angle x-ray scattering (SAXS) patterns were obtained for each GQD (fig. S9). GQD-to-GQD intermolecular distances for each GQDs are also indicated in fig. S9, using eq. S2. The parameters, q value at the peak positions of SAXS pattern (*q*^max^) and GQD-to-GQD intermolecular distance (*L)* values (see "Experimental determination of GQD-to-GQD intermolecular distance" section of Supplementary Text for details), for the series of GQDs are listed in table S4. We evaluated intermolecular distances of GQDs ranging from *q* = 0.5 nm^−1^ (*L* = 12.6 nm) to *q* = 5 nm^−1^ (*L* = 1.26 nm), because the range is known to mainly reveal particle-particle interactions (*53*) as well as exclude the particle size and interatomic contributions in the wide-angle regions. All GQDs showed multiple-peak nature, which is the characteristic property of layered structure (*54*) belonging to π-π stacked dimer, trimer structures of GQDs. The smallest intermolecular distance between GQDs is evaluated by the *L*_1_ value (table S4), increased from 1.49 nm for pristine GQD to 1.68, 1.85, and 2.33 nm for BA_1_-GQD, BA_2_-GQD, and TPE_2_-GQD, respectively. Likewise, the long-period patterns of corresponding GQDs followed the analogous tendencies: 4.03 nm (*L*_3_ of pristine GQD), 5.71 nm (*L*_5_ of BA_1_-GQD), 8.27 nm (*L*_3_ of BA_2_-GQD), and 9.67 nm (*L*_3_ of TPE_2_-GQD). (i) From comparison between BA_1_-GQD (*L*_1_ = 1.68 nm) and BA_2_-GQD, carbonyl-substituted BA_2_-GQD (*L*_1_ = 1.85 nm) gives more steric hindrance to GQDs than only carboxyl-substituted BA_1_-GQDs. Furthermore, (ii) from BA_2_-GQD and TPE_2_-GQD, as the ligands become bulkier, both the smallest intermolecular distance and long-period patterns shifted into longer distance. These speculations give experimental evidence that GQD-to-GQD intermolecular distance can be increased by not only substitutions of ligands with different chemical environment but also introduction of bulkier rotor molecules.

### PL mechanisms of GQDs

**Fig. 4.**
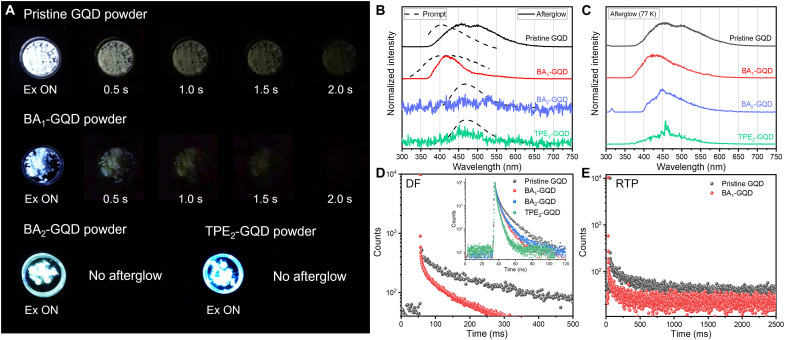
Afterglow properties of GQD powders with selective functionalization. Afterglow properties of GQDs with selective functionalization. (**A**) Real images of PF and RT afterglow of GQDs with respect to elapsed time. (**B**) Corresponding PF and RT afterglow spectra and (**C**) 77 K afterglow spectra of GQDs. (**D**) TCSPC decay of DF at λ_em_ = 454 nm for pristine GQDs and λ_em_ = 416 nm for BA_1_-GQDs, with TCSPC decay of PF of GQDs at their λ_em_ maximum (inset). (**E**) RTP at λ_em_ = 500 nm for pristine GQDs and λ_em_ = 512 nm for BA_1_-GQDs. No RTP decay was observed for BA_2_-GQDs or TPE_2_-GQDs.

To grasp the underlying mechanisms of AIE-active enhancement in solid-state emission, the afterglow properties of GQD powders were analyzed (Fig. 4) (*19*, *29*). Figure 4A shows digital images of the room temperature (RT) afterglows of various GQDs with respect to elapsed times until 2.0 s. Pristine GQDs and BA_1_-GQDs exhibit afterglow properties without embedding into any triplet-exciton stabilizing matrices (*19*, *55*, *56*). Moreover, even for similar blue prompt fluorescence (PF) for AIE-active GQDs, BA_2_-GQDs and TPE_2_-GQDs do not exhibit afterglow properties. As shown in Fig. 4A, the naked-eye–detectable white afterglow of pristine GQD powder at RT lasts for ~4.0 s (movie S1) in which the peaks appear at 454 and 500 nm (Fig. 4B). RT afterglow can also be detected from BA_1_-GQD powder (Fig. 4A); however, the emission is short-lived (lasting for 1.0 s) compared to that of pristine GQDs, with a blueshifted emission peak at 416 nm (Fig. 4B). BA_2_-GQD and TPE_2_-GQD powders, on the other hand, do not show any detectable afterglow, as seen in Fig. 4 (A and B), indicating that, despite the similar blue emission, GQDs undergo different processes in emissions. Long-lived afterglows result from radiative recombination of triplet excitons, and these RT afterglow origins (e.g., RTP or TADF) can be unveiled by the following parameters (*19*): (i) the peak wavelength between PF and afterglow and (ii) the thermal dependencies of afterglow. The low-temperature (77 K) afterglow helps us understand the RT afterglow origin because the triplet exciton is known to be stabilized as the temperature drops. Afterglow of pristine GQDs at 77 K (Fig. 4C) also appears at almost identical wavelengths (i.e., 454 and 500 nm) accompanied by an overall increase in the PL intensity of afterglow. However, with respect to their RT counterparts, at 77 K, the afterglow peak of BA_1_-GQDs is retained at 416 nm with a lower PL intensity; in addition, shoulder peaks at 512 nm emerge at the same time (Fig. 4C). According to Fig. 4C, although the RT afterglows of BA_2_-GQDs and TPE_2_-GQDs are almost undetectable, their afterglow properties are recovered at 77 K, with peaks appearing at 450 and 449 nm, respectively.

To determine whether the afterglow type is RTP or TADF, a temperature-dependent comparison of the afterglow intensity and time-resolved emission spectra (TRES) (*57*) was conducted by varying the gate delay time. The emissive origins of powder afterglows were compared with those of monomers (fig. S10), including the PF spectra of each GQD and the afterglow spectra in which the results are summarized in table S5. T_1_ state of pristine GQDs in monomer was assigned as a peak of fig. S10A, 441 nm (= 2.81 eV); likewise, T_1_ state of BA_1_-GQDs (fig. S10B), BA_2_-GQDs (fig. S10C), and TPE_2_-GQDs (fig. S10D) in monomer was assigned as 432 nm (= 2.86 eV), 457 nm (= 2.71 eV), and 500 nm (2.48 eV), respectively. As seen in fig. S11A and table S5, the PF peak wavelength of pristine GQDs is 402 nm (*S*_1_ = 3.08 eV), and their RT afterglow at 454 nm (T_1_ = 2.70 eV) and 500 nm (T_1_* = 2.47 eV) with an increase in intensity at 77 K demonstrates that the afterglow is caused by phosphorescence (RTP). On the other hand, at 77 K, the PF peak wavelength for BA_1_-GQDs is 416 nm (*S*_1_ = 3.10 eV), while the afterglow wavelength is almost the same at 416 nm (T_1_ = 2.98 eV), albeit with a reduction in intensity, which coincides with an increase in intensity at 512 nm (T_1_* = 2.42 eV). The RT afterglow of BA_1_-GQDs shows representative TADF behavior due to (i) a small singlet-triplet energy gap (Δ*E*_ST_) of 0.17 eV that can induce reverse ISC (RISC) at RT with prolonged emission [i.e., delayed fluorescence (DF)] and (ii) enhanced intensity in DF at 416 nm with increasing temperature, followed by a decrease in phosphorescence (512 nm) (*56*), consistent with the observation in fig. S11B. These opposite behaviors of these two peaks to thermal response correspond to the TADF process of S_0_→S_n_→T_n_→S_1_→S_0_. TRESs with various gate delay times support the abovementioned rationales more precisely. According to fig. S12A, pristine GQDs show an instant decrease in the PF peak at 402 nm (*S*_1_), and their peak is redshifted to 454 nm (T_1_) after a 1-μs delay. After 250 μs, the intensity of 500 nm (T_1_*) starts to increase, and, after 500 μs, the PF peak decays almost completely. This result implies that a 500-nm RTP has more prolonged lifetimes, which belongs to strong intermolecular interactions between pristine GQDs from π-π stacked aggregates (*58*, *59*). Furthermore, for BA_1_-GQDs in fig. S12B, the PF peak at 400 nm quickly diminished, and a 416-nm DF peak was observed after 1 to 8000 μs. The RTP component at 512 nm also increased; however, it decayed after 1000 μs, implying that the RISC process quenches the RTP and promotes DF after 1000 μs, supporting the TADF mechanism behind the BA_1_-GQDs (fig. S12B) (*56*, *57*). Even with an analogous increase in afterglow intensity at 77 K as in pristine GQDs, the emission in BA_2_-GQDs and TPE_2_-GQDs is primarily fluorescence because the RT afterglows are negligible for these GQDs. It should be noted that the appearance of afterglow at 77 K for BA_2_-GQDs and TPE_2_-GQDs could be due to a reduction in vibrational dissipation that acts as internal conversion, resulting in an overall increase in fluorescence rate (*k*_fl_) even after time has passed. Triplet emissions of GQDs can be realized in the blue emission region—blue RTP for pristine GQDs at 454 nm along with 500 nm for dual emission and blue TADF for BA1-GQDs at 416 nm (fig. S8D), which have rarely been reported for GQDs without inclusion of any matrices or host materials.

On a series of GQD powders, time-correlated single-photon counting (TCSPC) measurements were performed to characterize the decay landscapes. First, the radiative recombination rate (*k*_r_) and nonradiative recombination rate (*k*_nr_) of PF were calculated using eqs. S3 and S4 after obtaining the average decay lifetimes (<τ>) of GQDs in various states using eq. S5. From tables S6 and S7 derived from the TSCPC results from fig. S13 and Fig. 4D (inset), *k*_r_ and *k*_nr_ were compared to investigate the difference in photophysical behaviors between solution and powder. Pristine GQDs showed a *k*_r_ of 1.397 × 10^7^ s^−1^ and *k*_nr_ of 19.14 × 10^7^ s^−1^ in solution (table S6), which was changed to a *k*_r_ of 0.481 × 10^7^ s^−1^ and *k*_nr_ of 18.77 × 10^7^ s^−1^ in powder. The reduced *k*_r_ in powder compared to *k*_r_ in solution is due to the ACQ phenomena that quenches the PF from GQDs by activation of relatively low-PLQY RTP with increased ISC. On the other hand, *k*_r_ increased from 0.588 × 10^7^ s^−1 ^and 1.394 × 10^7^ s^−1^ in solution (table S6) to 1.099 × 10^7^ s^−1^ and 3.352 × 10^7^ s^−1^ in powder (table S7) for BA_1_-GQDs and BA_2_-GQDs, respectively. *k*_nr_ for the same GQDs showed sufficiently decreased values from solution to powder, i.e., 17.78 × 10^7^ s^−1^ and 25.94 × 10^7^ s^−1^ to 12.81 × 10^7^ s^−1^ and 18.14 × 10^7^ s^−1^. These opposite tendencies in BA_1_-GQDs and BA_2_-GQDs from pristine GQDs are consistent with AIE molecules having an RIR mechanism (*49*), showing a decrease in internal conversion that acts as nonradiative processes in their solid states. For TPE_2_-GQDs, *k*_r_ is greatly increased in the solid state compared to the solution state by 23-fold with an increase in *k*_nr_, which may be ascribed to unwanted intermolecular interactions between TPE_2_-GQDs.

Figure 4D shows the decay curve regarding the fluorescence (inset, S_0_→S_n_→S_0_) and DF (S_0_→S_n_→T_n_→S_1_→S_0_) of each GQD. Unlike BA_2_-GQDs and TPE_2_-GQDs, which exhibits only PF, pristine GQDs and BA_1_-GQDs consist of a fast decay component (about nanoseconds), as shown in the inset of Fig. 4D and table S7, and a delayed decay component (about milliseconds), and these results match well with those of Fig. 4 (A and B), as only these two GQDs have distinctive emission origins of PF and afterglow. The average afterglow lifetime at the nearest DF for pristine GQDs (454 nm) and BA_1_-GQDs (416 nm) is calculated to be 240.9 ms and 114.8 ms, respectively (table S8), showing good agreement with not only the TRES results in fig. S12 but also the shortened decay curves for BA_1_-GQDs compared to pristine GQDs shown in Fig. 4D. Likewise, the average lifetimes for direct emission from RTP at peak wavelengths of 500 and 512 nm for pristine GQDs and BA_1_-GQDs are 326.4 and 108.5 ms (table S8), respectively, as shown via the shortened decay for BA_1_-GQDs shown in Fig. 4E. The obtained lifetime values are in good agreement with the digital afterglow images in Fig. 4A, which show that the overall RT afterglows of BA_1_-GQDs are shorter than those of pristine GQDs and again corroborate that 454-nm emission for pristine GQDs has RTP properties.

Table S9 is a summary of photophysical parameters of GQDs or carbon dots (CDs) from previous literatures in which solid-state PLQY and their emission mechanisms are specified. When it comes to GQDs made using the top-down method, most literature does not specifically report solid-state PLQY, and even the PLQY in a well-dispersed monomer state is around 10%. BA_2_-GQDs and TPE_2_-GQDs showed relatively high PLQY comparing to those of GQDs in matrices (entries 1 to 5) and bottom-up–based CDs without matrices (entries 6 to 9). Moreover, blue emission having with various emission nature, i.e., RTP, TADF, and fluorescence, can be achieved herein only by controlling CT states that are different from bare CDs having inherent triplet-stabilizing random sp^3^ networks (entries 6 to 9) or matrices exhibiting the analogous effects (entries 10 to 11).

### Theoretical calculations

**Fig. 5.**
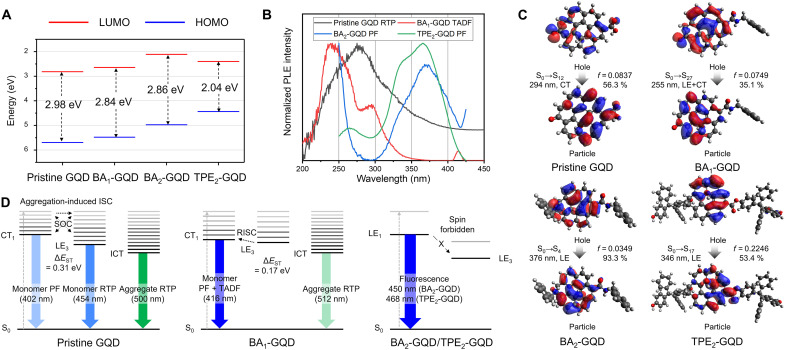
Calculated molecular orbitals and PL mechanisms for various GQDs. (**A**) Highest occupied molecular orbital (HOMO) and lowest unoccupied molecular orbital (LUMO) levels of GQDs. (**B**) PLE spectra for afterglows for GQDs. (**C**) NTOs of singlet states for various GQDs at PLE maxima. (**D**) Proposed mechanisms for the photophysical phenomena of GQDs in powder.

DFT calculations were performed to further elucidate the emission mechanisms of several GQDs in detail (Fig. 5 and Materials and Methods). Figure 5A represents the highest occupied molecular orbital (HOMO) and the lowest unoccupied molecular orbital (LUMO) levels of GQDs. The HOMO level of AIE-active GQDs is higher than that of the pristine GQDs, mostly due to the introduction of electron-donating groups in BA and TPE-DOH, such as amine and hydroxyl groups, helping to delocalize holes more easily. Reduction in the HOMO-LUMO gap was observed for all GQDs with respect to pristine GQDs because of the increase in the push-pull effect induced between the amine- or hydroxyl-based electron donating group and carbonyl- or carboxyl-based electron withdrawing group, which contributes to narrowing the HOMO-LUMO gap (*60*). The HOMO-LUMO gap found experimentally from the GQD powder PL [Fig. 2 (E to H)] follows trends similar to those calculated, showing that the chemical configurations used in computations are adequate for explaining our results. Along with frontier orbital considerations, the absorption spectra of GQDs in solution (monomer) and powder were considered to scrutinize the ground state interactions. Pristine GQDs in solution show shoulder peaks at ~250 and ~280 nm, which were assigned as the characteristic π-π* transition of the sp^2^ carbon structures of GQDs; this was consistent with previous studies (fig. S11A) (*10*, *18*, *61*). For all GQDs in solution, prominent peaks at 305 to 315 nm and 325 to 335 nm may belong to the n-π* transition of C═O (pristine GQD, BA_1_-GQD) (*62*) or C─N (BA_2_-GQD), C─O─C (TPE_2_-GQD), and ─COOH (pristine GQD) (*63*), ─CONH─ (BA_1_-GQD, BA_2_-GQD), and ─COO─ (TPE_2_-GQD), respectively (fig. S14, A to D). The absorbance of the pristine GQD and BA_1_-GQD powders increased throughout the whole wavelength range, and a distinctive shoulder peak at 375 nm appeared, which cannot be observed in the solution for both GQDs (fig. S14, A and B). This overall increase of absorbance in GQD powders connotes strong intermolecular interactions between GQDs, and, in particular, the new peak at 375 nm can be assigned as intermolecular CT absorption (*64*, *65*) in powder. As the GQDs packed close enough, their photophysical properties become different to those of monomers, indicating the possibilities for strong orbital interactions between GQDs. In contrast, compared to the solution, BA_2_-GQD powder showed almost similar absorption, indicating the effective suppression of intermolecular interactions.

Comparison of normalized PL excitation (PLE) spectra between solution (monomer) and powder helps to understand the PL origins of GQDs in each state (*58*, *66*). According to fig. S15 (A to D), the pristine GQDs and BA_1_-GQDs exhibited different origins for PF in the monomer and powders. The singlet characteristic ^1^(π-π*) LE transition at approximately 250 to 280 nm serves as an origin for PF in both GQDs in solution, and ^1^(π-π*) LE with a shoulder peak of the intermolecular CT transition appears as an origin for the PF triplet emission at ~375 nm in pristine GQDs. In powder, the PF of S_1_→S_0_ for both GQDs (λ_em_ = 402 nm for pristine GQDs, 416 nm for BA_1_-GQDs) is mainly dominated by the ^1^(n-π*) CT transition at ~350 nm, which was the major origin for phosphorescence in monomers. It is crucial to point out that PLE peaks at 375 nm prevailed for T_1_→S_0_ and T_1_*→S_0_ emission in both GQDs in powder, meaning that totally different energy levels, i.e., intermolecular CT bands, contribute to triplet emissions in powders, which also conformed with the previous absorption spectra in fig. S14 (A and B). PF origins for both monomer and powders share the same origins at ~250 and ~370 nm and at ~250 nm and 320 to 370 nm for BA_2_-GQD and TPE_2_-GQDs, respectively (fig. S15, E to H). Sufficient steric hindrance induced by rotor molecules at the edges inhibits the intermolecular interactions and helps to maintain monomer characteristics even in powder for both BA_2_-GQDs and TPE_2_-GQDs. Conversely, we carefully reasoned that the PF characteristics of GQD powders with afterglow properties are different from those of monomers due to the presence of intermolecular interactions between GQDs.

Figure 5B demonstrates the correlation between theoretical singlet/triplet absorption calculations and PLE spectra for representative emission to determine the most likely transitions involved in afterglow emission. Along with Fig. 5B, fig. S16 helps to understand the overall outline for afterglow origins in detail. T_1_→S_0_ and T_1_*→S_0_ RTP of pristine GQDs stem mostly from mixed states of ^3^(π-π*) LE at 260 nm and ^1^(π-π*) CT at 300 nm, and additional peaks at 320 nm appeared as a result of strong intermolecular interactions (fig. S16A). For BA_2_-GQDs (fig. S16B), all the afterglow components from TADF and RTPs belong to apparent mixing between ^3^(π-π*) LE at 250 nm, which are dominant origins for monomer (fig. S16C), ^1^(n-π*) CT at 300 nm, and even intermolecular CT at 350 nm. Taken the results together, we deduced that multiple activation channels [i.e., ISC by spin-orbit coupling (SOC)] are present for TADF and RTPs in BA_1_-GQD powders, and PLE of TADF shows similar characteristics of monomer emission. From fig. S16 (C and D), although BA_2_-GQDs and TPE_2_-GQDs have a negligible afterglow, the monotonous PLE peak of ^1^(π-π*) LE at 280 nm can also be found as a part of the ^1^(π-π*) LE peaks in monomer and powder PF (fig. S15, E to H), indicating the fluorescence characteristics, even for the afterglows.

Of these theoretical transitions, the transitions with the highest oscillator strength (*f*) at PLE peaks is provided in Fig. 5C in the form of a natural transition orbital (NTO), which can well-describe the intramolecular CT. The most likely transition for pristine GQDs engaged in RTP is related to the ^1^(n-π*) transition (294 nm), which is theoretically equivalent to the S_0_→S_12_ transition, as pictured in Fig. 5C. This transition coincides with the strong intramolecular CT depicted as a ^1^(n-π*) singlet transition because the apparent CT from the n orbitals of the C═O group to π electrons adjacent to the ─COOH group that is originally deficient in holes. Likewise, the most likely transitions promoting BA_1_-GQD TADF are related to the ^1^(n-π*) transition (255 nm), and the CT mixed with LE characteristics is also verified with CT from n electrons at C═O edge sites to π electrons in the basal plane and retained LE π electrons near the C═O groups (S_0_→S_27_, Fig. 5C). The PLE of BA_2_-GQDs and TPE_2_-GQDs shows a LE consisting of ^1^(π-π*) characteristics at 375 nm (Fig. 5B), corresponding to the S_0_→S_2_ transition and S_0_→S_17_, theoretically for BA_2_-GQDs and TPE_2_-GQDs. El-Sayed’s rule states that (i) SOC is possible between CT states with singlet characteristics (^1^CT) and LE states with triplet characteristics (^3^LE) and vice versa, and (ii) SOC is forbidden between the same characteristics with different spin states (e.g., ^1^LE and ^3^LE, and ^1^CT and ^3^CT) (*67*). On the basis of this rule, we can determine that, while similar blue PFs emerge in all four GQDs, GQDs with strong ^1^CT characteristics, such as pristine GQDs and BA_1_-GQDs, allow SOC to ^3^LE states to form triplet-mediated RTP or TADF. In the case of BA_2_-GQDs and TPE_2_-GQDs, because of the absence of n orbitals lying perpendiculr to π orbitals due to the substitution of C═O into the amine group, SOC between ^1^(n-π*) and ^3^(π-π*) may be prevented, reducing *k*_ISC_ and enhancing *k*_fl,_ estimated as *k*_r_ herein. As a result, unlike pristine GQDs and BA_1_-GQDs, BA_2_-GQDs and TPE_2_-GQDs exhibit no afterglow properties in their powder forms and show relatively high Φ_F,powder_. The overall landscape of the energy diagram for each type of GQD is outlined in Fig. 5D. The experimental values of Δ*E*_ST_ for each GQD were obtained by measuring the afterglow spectra of GQDs in solution at 77 K (table S5). The Δ*E*_ST_ values of the powder (0.31 eV) showed a slightly reduced value compared to that of the monomer (0.39 eV) because of strong intermolecular orbital interactions between GQDs from multimer states, which can split singlet/triplet energy levels to form a cascade-like energy landscape, increasing the ISC channel, denoted as aggregation-induced ISC (*68*). We also speculated from the behaviors of BA_1_-GQDs in Fig. 5B and figs. S15 and S16 that, in BA_1_-GQDs, monomer TADF is the dominant mechanism because the sufficiently small Δ*E*_ST_ = 0.24 eV in monomer and Δ*E*_ST_ = 0.17 eV of powders facilitate RISC in RT, and this RISC is even possible for spin mixing between intermolecular CT states (350 nm) of multimers and various monomer LE states (*66*) with the help of aggregation-induced ISC. Ultimately, for BA_2_-GQDs and TPE_2_-GQDs, most of their transitions are composed of ^1^LE characteristics, which were proven experimentally by PLE and theoretically by NTO analysis. The results validate the lack of intra- or intermolecular spin mixing, i.e., suppressing ISC, resulting in an increase in *k*_fl_ of high QY up to 16.8% from pure LE states.

## DISCUSSION

In conclusion, we reported simple strategies for realizing solid-state emission from GQDs via (i) size reduction from 5 to 1 nm and (ii) selective edge functionalization using rotor molecules of BA and TPE-DOH. Reducing the physical size of GQDs from 5 to 1 nm successfully suppressed ACQ, with the exhibition of blue (~450 nm) emission and solid-state PLQY up to 2.5% without any treatment. Edge functionalization of rotor molecules endows GQDs with AIE characteristics, with stronger solid-state emission than solution emission, which further enhances the solid-state PLQY to 16.8% for TPE_2_-GQDs. Moreover, functionalized GQDs exhibit enhanced solid-state PLQY compared to that of pristine GQDs (BA_1_-GQDs, BA_2_-GQDs, and TPE_2_-GQDs exhibit 3.2-, 6.2-, and 6.7-fold increases, respectively), retaining their blue emission (450 to 468 nm). In addition, the emission behaviors of GQDs are converted from RTP and TADF to fluorescence with the substitution of C═O and COOH groups in GQDs, effectively suppressing ISC in both inter- and intramolecular manners. The transition of RTP and TADF to fluorescence of top-down–based GQDs in matrix-free conditions is first observed, which is crucial to boosting the PLQY of fluorescent materials to high quantum efficiency. Theoretical investigation also reveals that the carbonyl group of GQDs facilitates ISC through mixing of the ^1^CT and ^3^LE states with a small *k*_fl_, which is consistent with the experimental results. We believe that the GQDs on a 1-nm scale (less than 20 benzene rings) facilitate the molecular engineering method for organic molecules, enabling the selective edge functionalization of rotor molecules. This work will create new prospects for using GQDs in solid-state LEDs in the near future with the effective manipulation of various inter/intramolecular CT states of GQDs, revealed with improvements in FWHM and PLQY that are not yet understood in detail.

## MATERIALS AND METHODS

### Synthesis of 5-nm GQDs, 2-nm GQDs, and pristine GQDs

GQDs of different sizes were exfoliated with GICs, and the typical procedure for preparing GQDs is as follows. One gram of graphite (Bay carbon) and 15 g of potassium sodium tartrate tetrahydrate (KNaC_4_H_4_O_6_·4H_2_O) were physically ground on an agate mortar. The ground mixture was loaded in a vial and heated to 270°C for 24 hours to obtain swollen GICs. The GICs were dispersed in deionized water and exfoliated via ultrasonication for 3 hours to produce GQDs. As-exfoliated GQDs were separated with a 10,000-Da MWCO centrifugal filter, and the filtrate was consecutively dialyzed with 3500-, 1000-, and 500-Da dialysis bags. GQDs (5 nm) were collected as an aqueous solution inside a dialysis bag after 5 days of dialysis using 3500-Da MWCO. The dialysate of the 3500-Da MWCO dialysis bag was collected and dialyzed with a 1000-Da MWCO dialysis bag for 5 days, and 2-nm GQDs were collected as an aqueous solution inside the dialysis bag. The dialysate of the 1000-Da MWCO dialysis bag was collected and dialyzed with a 500-Da MWCO dialysis bag for 5 days, and pristine GQDs were collected as an aqueous solution inside the dialysis bag. GQDs were freeze-dried for 24 hours to obtain GQDs in a powder form. The molecular weight distributions of GQDs with different sizes are as follows: 5-nm GQDs (3500 to 10,000 Da), 2-nm GQDs (1000 to 3500 Da), and 1-nm GQDs (pristine GQD, 500 to 1000 Da).

### Synthesis of BA_1_-GQDs

Typical procedures for the synthesis of BA1-GQDs via carbodiimide cross-linker chemistry are as follows. Thirty milligrams of pristine GQD powder was dispersed in 30 ml of water (ca. 1 mg/ml), 100 mg of EDC-HCl [*N*-(3-dimethylaminopropyl)-*N′*-ethylcarbodiimide hydrochloride] and 110 mg of sulfo–*N*-hydroxysulfosuccinimide sodium salt were added, and the solution was stirred for 30 min at RT. In this step, the carboxylic acid groups of the pristine GQDs are converted into *N*-hydroxysulfosuccinimide ester, which can readily form amide bonds with amine derivatives. In the solution, 20 ml of ethanol solution (5 mg/ml) of BA was added and stirred for 24 hours at 100°C. Unreacted reactants were removed by 3 days of dialysis (500-Da MWCO), and the products were freeze-dried for 24 hours to obtain BA_1_-GQDs in a powder form.

### Synthesis of BA_2_-GQDs

Typical procedures for the synthesis of BA2-GQDs via reductive amination are as follows. Thirty milligrams of BA_1_-GQD powder was dispersed in 10 ml of acetic acid (ca. 3 mg/ml), 50 mg of pic-BH_3_ (2-methylpyridine borane complex) was added, and the solution was stirred at RT. In the solution, 20 ml of isopropyl alcohol solution (5 mg/ml) of BA was added and stirred for 72 hours at 50°C. Unreacted reactants were removed by 3 days of dialysis (500-Da MWCO), and products were freeze-dried for 24 hours to obtain BA_2_-GQDs in a powder form.

### Synthesis of TPE_2_-GQDs

The typical procedure for the synthesis of TPE_2_-GQDs via Steglich esterification is as follows. Ten milligrams of pristine GQD powder was dispersed in 30 ml of acetonitrile (ca. 0.3 mg/ml), 190 mg of EDC-HCl and 80 mg of 4-(dimethylamino)pyridine were added, and the solution was stirred for 30 min at RT. In the solution, 25 mg of TPE-DOH was added and stirred for 72 hours at 85°C. Unreacted reactants were removed by 3 days of dialysis (500-Da MWCO), and the products were freeze-dried for 24 hours to obtain TPE_1_-GQDs in a powder form. As-synthesized TPE_1_-GQDs were redispersed in 30 ml of THF (ca. 1 mg/ml), and 100 mg of NaBH_4_ (sodium borohydride) and 25 mg of TPE-DOH were added in the presence of 0.2 ml of aqueous HCl (37%). The solution was stirred for 72 hours at 85°C and dialyzed with 500-Da MWCO for 3 days, followed by freeze-drying for 24 hours to obtain TPE_2_-GQDs in a powder form.

### Characterization methods

TEM images were acquired using a Titan G2 Cube 60-300 operation at 80 kV. XPS data were collected using K-alpha (Thermo VG Scientific) XPS. ^13^C NMR spectra were obtained with an Avance Neo (Prodigy, Bruker Biospin) 600-MHz spectrometer. Pristine GQDs, BA_1_-GQDs, and BA_2_-GQDs were dispersed in deuterium oxide, and TPE_2_-GQDs were dispersed in d_6_-DMSO to conduct NMR measurements. FTIR spectra were collected using a Nicolet iS50 (Thermo Fisher Scientific Instrument) FTIR spectrometer. SAXS measurements of GQD powders were performed using Nanopix SAXS instrument (Rigaku) with Cu-target x-ray. Ultraviolet-visible (UV-vis) absorption was measured using a SolidSpec-3700 UV-vis/near-infrared spectrophotometer. PL and PLE analyses were performed using an F-7000 (Hitachi) fluorospectrophotometer. Afterglow spectra were obtained with an F-7000 using a 40-Hz chopper with a gate delay time of 1 ms. TRES were also obtained using the same equipment by varying the gate delay time from 1 to 8000 μs. PF, RTP, and TADF decay lifetimes were collected via TCSPC using Fluorolog3 (Horiba). The absolute PL PLQYs were measured using Quantaurus-QY C13534-11 (Hamamatsu). The space charge–limited current behavior of GQDs was assessed with current-voltage characteristics using a Keithley 2635A source meter unit.

### Computational details

Time-dependent DFT (TDDFT) calculations were performed on Gaussian 16 Rev. A03 program (*69*). The structures of various GQDs were optimized for their ground state (*S*_0_) at the DFT level of theory using Becke’s three-parameter exchange functional along with Lee Yang Parr’s correlation functional (B3LYP) using 6-31G(d) basis sets. The first 30 singlet (*S*_n_) and first 50 triplet (*T*_n_) excited states and their NTOs are considered on the basis of TDDFT methods.

## Supplementary Materials

### This PDF file includes:

Supplementary Text Figs. S1 to S21 Tables S1 to S9 Legend for movie S1 References
