# Supplementary material for: Aggregation-induced emission of matrix-free graphene quantum dots via selective edge functionalization of rotor molecules
Source: Sci Adv. 2023 Feb 17;9(7):eade2585. doi: 10.1126/sciadv.ade2585 (PMC9937574; doi:10.1126/sciadv.ade2585)
Supplement: Supplementary file 1 — Supplementary Text Figs. S1 to S21 Tables S1 to S9 Legend for movie S1 References [file sciadv.ade2585_sm.pdf]

Science Advances
AAAS

Supplementary Materials for

Aggregation-induced emission of matrix-free graphene quantum dots via selective edge functionalization of rotor molecules

Sukki Lee *et al.*

Corresponding author: Seokwoo Jeon, jeon39@kaist.ac.kr, jeon39@korea.ac.kr

Sci. Adv. **9**, eade2585 (2023)
DOI: 10.1126/sciadv.ade2585

The PDF file includes:

Supplementary Text
Figs. S1 to S21
Tables S1 to S9
Legend for movie S1
References

Other Supplementary Material for this manuscript includes the following:

Movie S1

Supplementary Text

Experimental determination of energy levels

Singlet energy level (S_1) of various GQDs were measured as PL peak maxima of prompt fluorescence having highest PLQY for both solutions (monomer) and powders. Triplet energy levels (T_1) of various GQDs in monomer were measured as PL peak maxima of afterglow with highest PL intensity in solution at 77 K (λex = 285 nm). Triplet energy levels (T_1, T_1*) of various GQDs in powder were measured as PL peak maxima of afterglow with highest PL intensity in powders at 300 K (λex = 285 nm). Singlet-triplet energy gap (ΔE_{ST}) is calculated as energy difference between S_1 and T_1 level.

Definitions of various photophysical paramter

FWHM of GQDs in "good" solvents (f_{THF} = 0) is denoted as $\Delta\lambda_{soln}$, and that in "poor" solvents (f_{THF} = 90) is denoted as $\Delta\lambda_{,agg.}$ Absolute PLQYs in powder is denoted as $\Delta\lambda_{,powder.}$

Absolute PLQY of GQDs in "good" solvents (f_{THF} = 0) is denoted as $\Phi_{F,soln}$, and that in "poor" solvents (f_{THF} = 90) is denoted as $\Phi_{F,agg.}$ Absolute PLQYs in powder is denoted as $\Phi_{F,powder.}$ α_{AIE} value of GQD is defined using Supplementary Equation (1):

$$\alpha_{AIE} = \frac{\Phi_{F,powder}}{\Phi_{F,soln}} \quad (1)$$

Experimental determination of GQD-to-GQD intermolecular distance

Small angle X-ray scattering (SAXS) techniques to reveal the interparticle distance of each GQD. GQD-to-GQD intermolecular distance (L) is derived from the Supplementary Equation (2) that can be found from elsewhere (*70*):

$$L\ (nm) = \frac{2\pi}{q^{max}} \tag{2}$$

where q^{max} is the peak positions of SAXS pattern of each GQDs with respect to scattering vector q (nm^{-1}).

Calculation details on various photophysical parameter

Radiative recombination rates (k_r) non-radiative recombination rates (k_{nr}) for GQDs are calculated with Supplementary Equation (3-4):

$$k_r = \frac{\Phi_F}{< \tau >} \tag{3}$$

$$k_{nr} = \frac{1}{< \tau >} - k_r \tag{4}$$

Average decay lifetimes ($<\tau>$) were calculated from each lifetime τ_i and its relative amplitude A_i obtained by multi-exponential fitting using Supplementary Equation (5):

$$< \tau > = \frac{\sum_i A_i \tau_i^2}{\sum_i A_i \tau_i} \tag{5}$$

Supplementary demonstration for aggregation-induced ISC

Along with the El-Sayed rule, spin can also be converted into singlet or triplet (SOC); as a result, ISC can occur with the help of additional thermal energy (ΔE_{ST}). This means that if we reduce the energy gaps between spin-allowed transitions in El-Sayed's rule, such as ^{1}LE↔^{3}CT and ^{1}CT↔^{3}LE, spin can even more likely be converted with the help of thermal energies. In this case, aggregations of GQDs, resulting from strong intermolecular interactions, increased the SOC channel by splitting the energy levels, which is called aggregation-induced ISC. This concept can be corroborated by the observations that triplet-mediated emissions, such as RTP and TADF, activated only in aggregated form, not in the monomer, which herein can be identified by the PL behaviors of pristine GQDs and BA_1-GQDs.

Characterization method for charge transport properties of GQDs

Charge transport properties of GQDs were measured via space charge limited current (SCLC) analysis using each GQD as an active layer with following device structure, ITO /PEDOT:PSS/TFB/GQD/LiF/Al. All layers except electrodes are fabricated by layer-by-layer spin-coating of each material in 4 mg/mL solution with 5000 rpm, 30 sec, baked at 150 °C for 30 min. Current density-voltage (J-V) characteristics of each GQD are divided into four charge transport regions: (i) ohmic region ($J \propto V$), (ii) trap SCLC region ($J \propto V^2$), (iii) trap-filled limited (TFL) region ($J \propto V^n$, $n > 2$), and (iv) SCLC region ($J \propto V^2$) in the absence of trapping. Defect density can be calculated with the onset voltage of TFL region is denoted as V_{TFL}, and which follows the Supplementary Equation (6) below:

$$V_{TFL} \approx \frac{e n_t L^2}{2\varepsilon_0 \varepsilon_r} \quad (6)$$

where n_t corresponds to trap-state density at the onset voltage, e the electronic charge, L the thickness of the active material, ε_0 the permittivity of free space, and ε_r the dielectric constant. Defect-mediated charge transport properties are evaluated via comparison on the value n_t of each GQD.

In the SCLC region, space charge mediates the charge transport characteristics thus current density follows Mott-Gurney law that can be expressed as the Supplementary Equation (7):

$$J_{SCLC} = \frac{9}{8}\varepsilon_0\varepsilon_r\mu\frac{V^2}{L^3} \quad (7)$$

where μ is the carrier mobility. Influence of space charge on charge transport at this region is assessed by J_{SCLC} and μ. In Supplementary Equation (6) and (7), it is assumed that ε_0 and ε_r is identical for all GQDs."

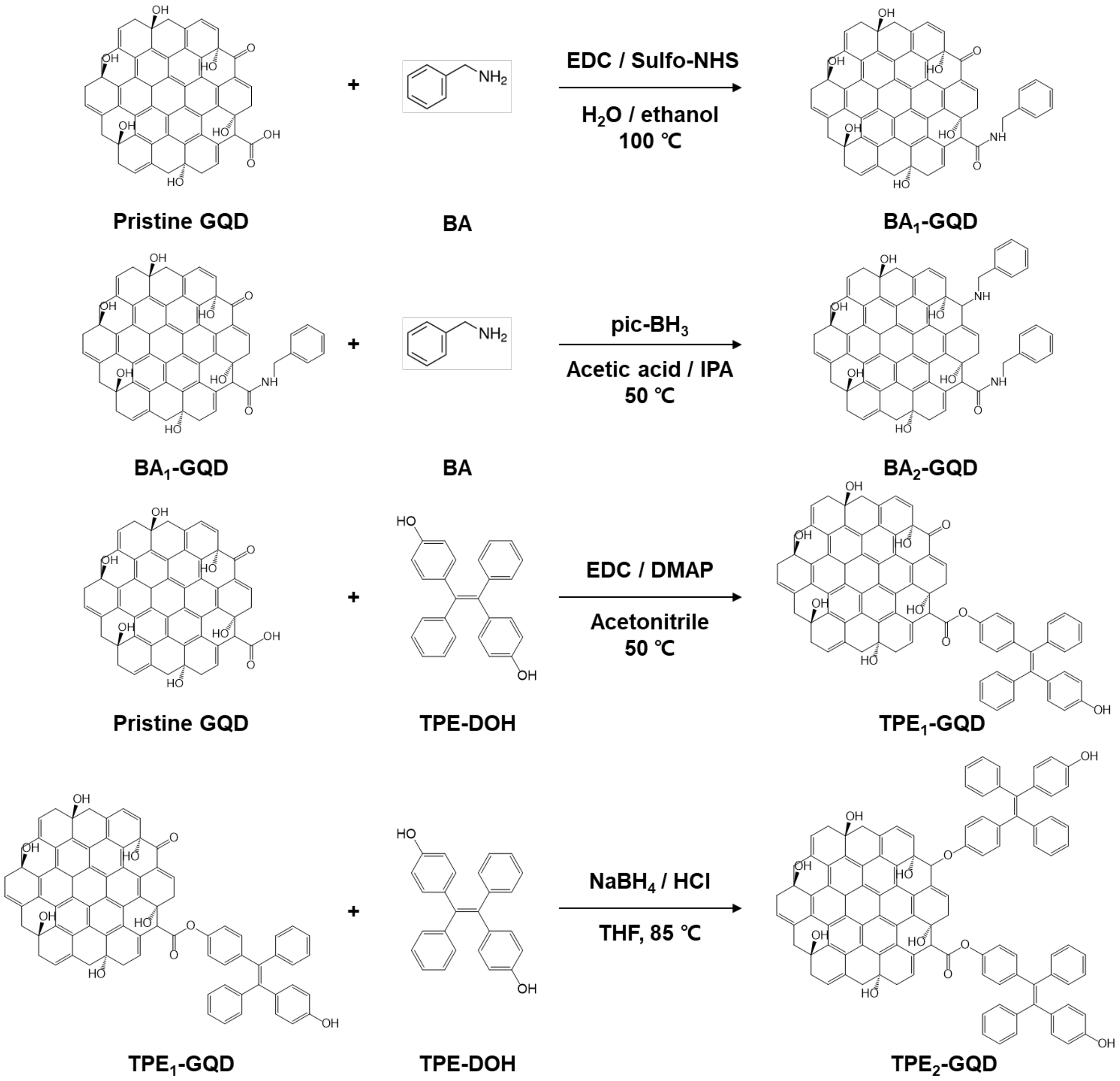

Fig. S1. Synthetic routes to BA_1-GQD, BA_2-GQD, and TPE_2-GQD

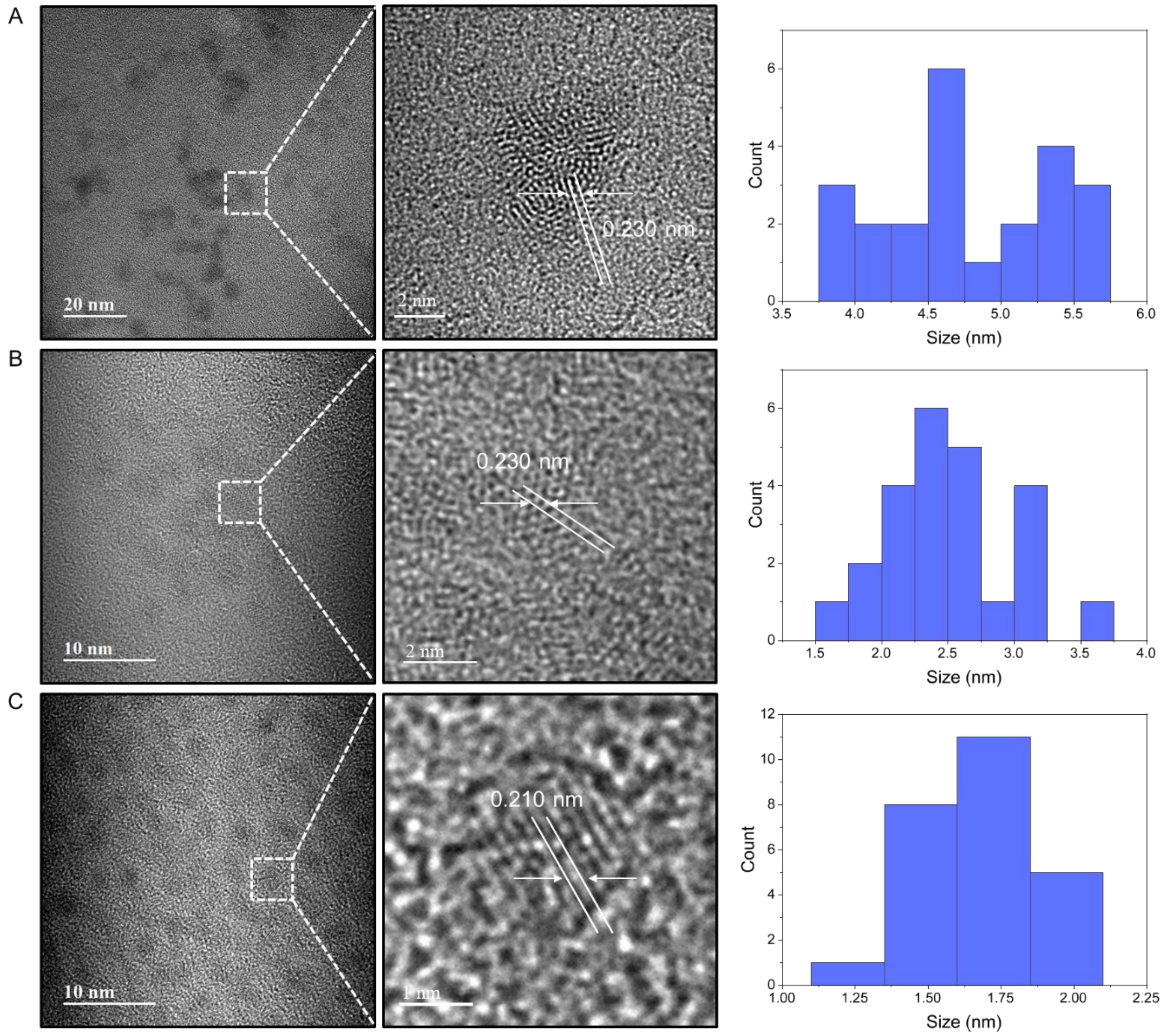

Figure S2. Size assessment of GQDs. Transmission electron microscope (TEM) images (left and middle) of GQDs with different sizes and their relative size distributions (right). (**A**) 5 nm (**B**) 2 nm (**C**) 1 nm.

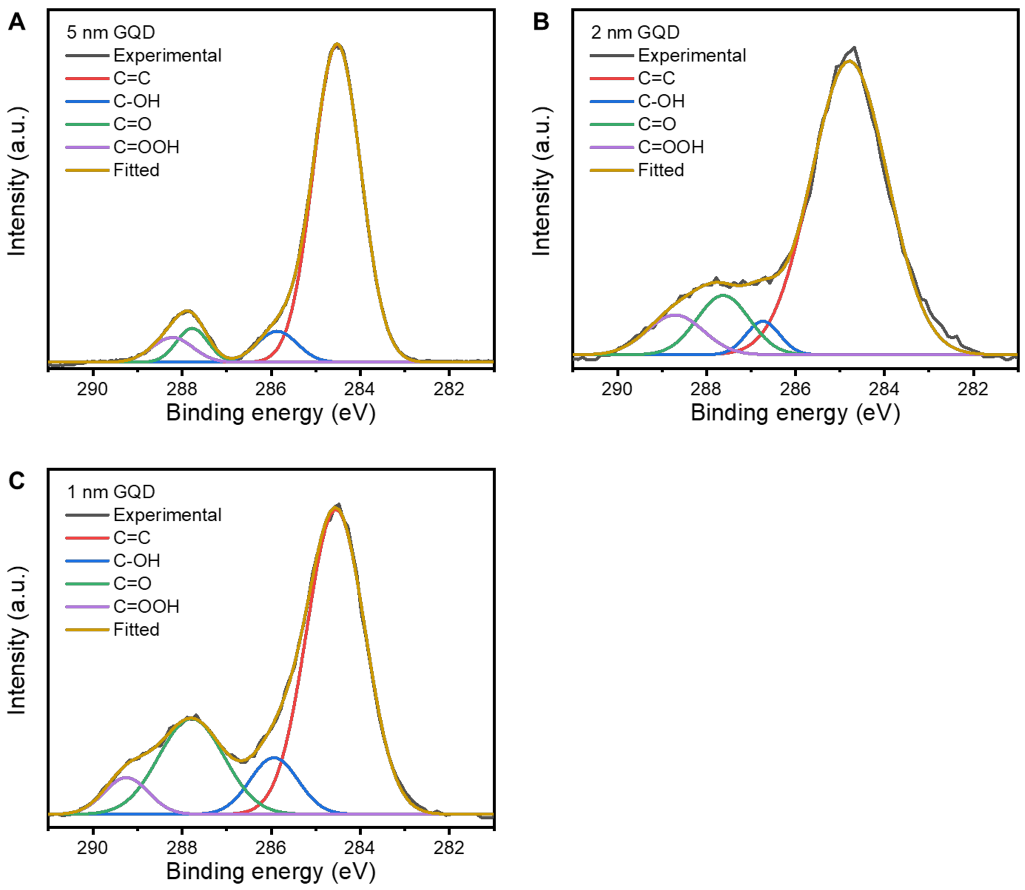

Fig. S3. XPS C1s spectra of GQDs with different sizes. (**A**) 5 nm GQD (**B**) 2 nm GQD (**C**) 1 nm GQD (pristine GQD).

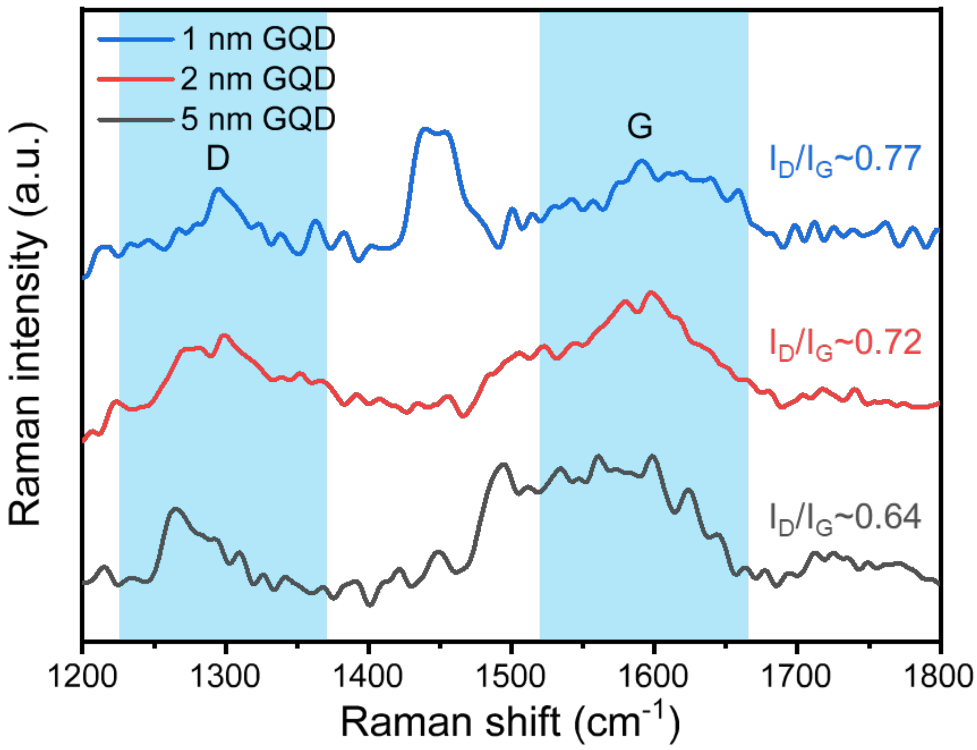

Fig. S4. Raman spectra of GQDs with different sizes.

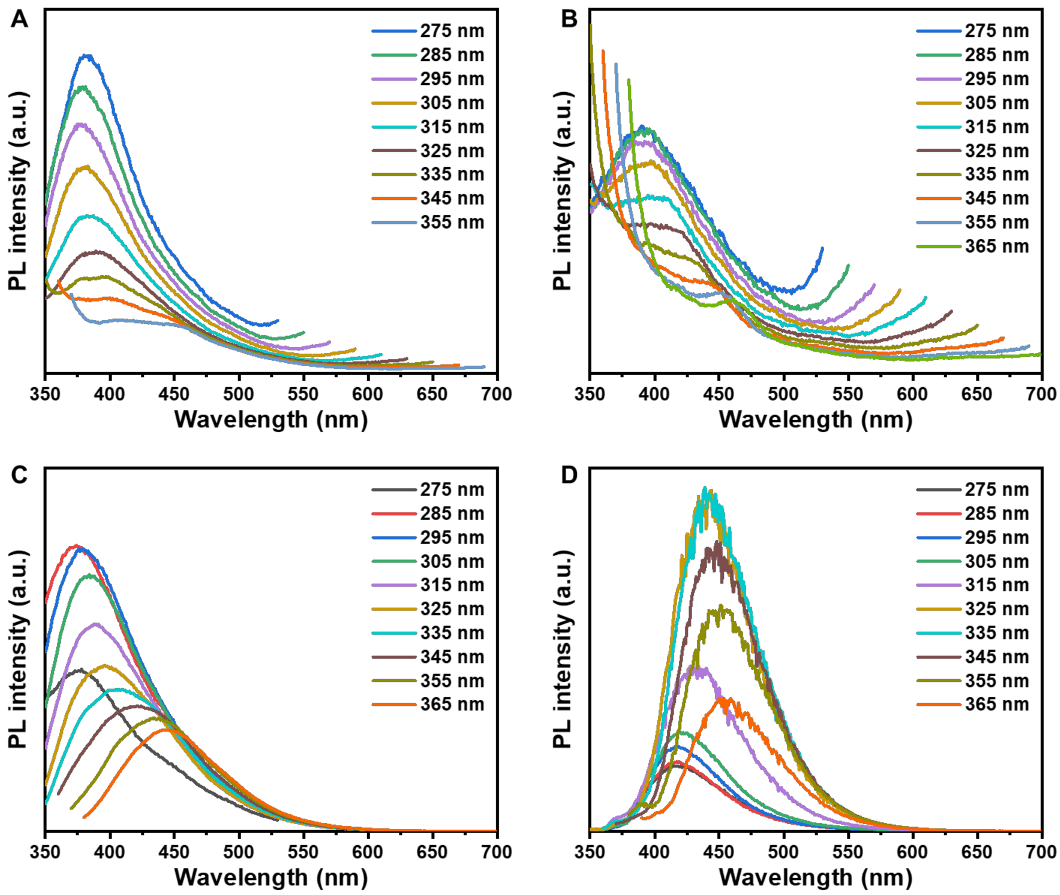

Fig. S5. PL spectra of various GQDs (f_{THF} = 0) with different wavelengths. (**A**) Pristine GQD (**B**) BA_1-GQD (**C**) BA_2-GQD (**D**) TPE_2-GQD

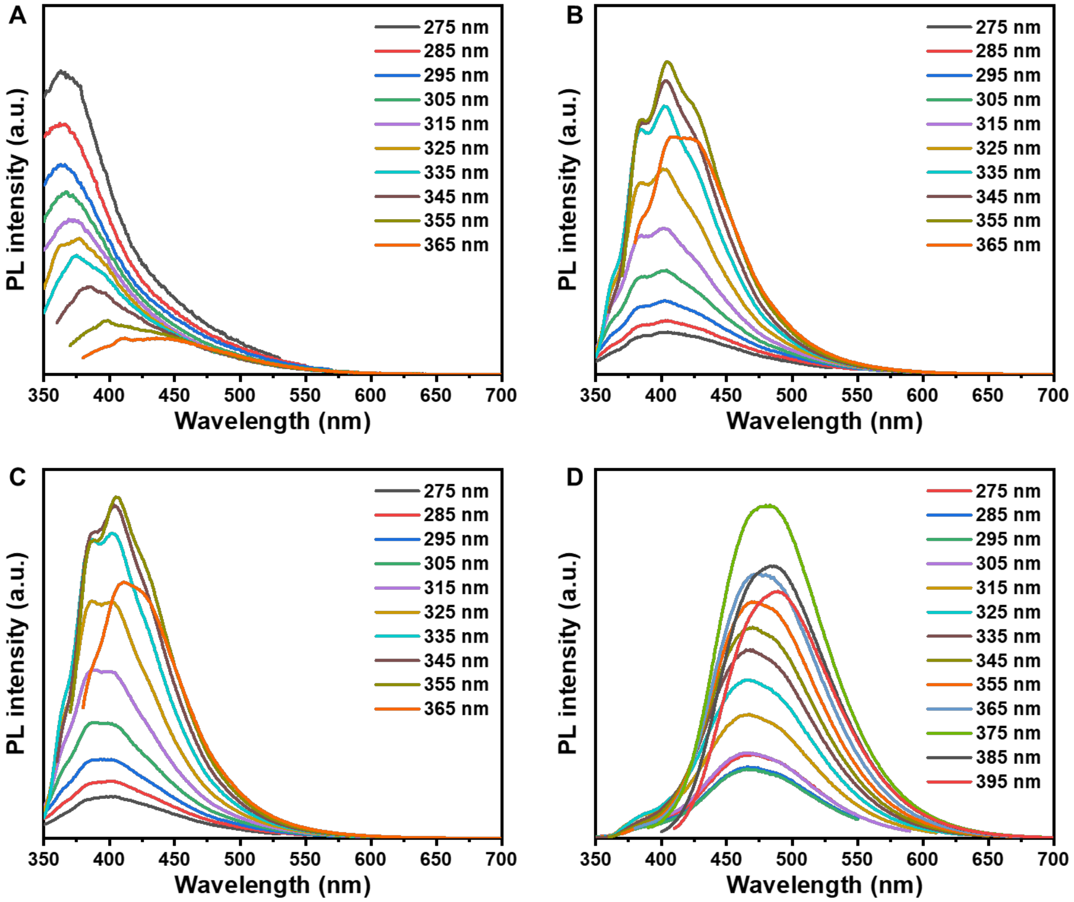

Fig. S6. PL spectra of various GQDs at f_{THF}=90 with different wavelengths. (**A**) Pristine GQD (**B**) BA_1-GQD (**C**) BA_2-GQD (**D**) TPE_2-GQD

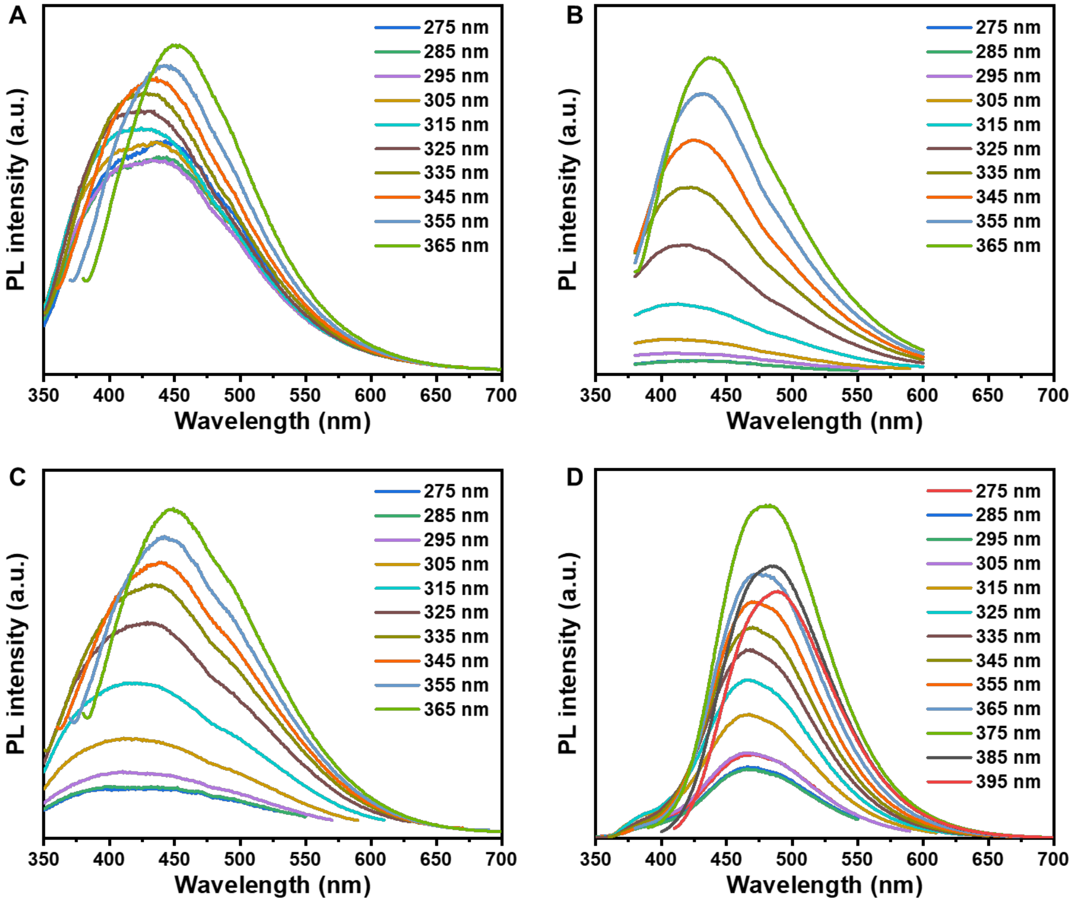

Fig. S7. PL spectra of various GQDs in powder with different wavelengths. (**A**) Pristine GQD (**B**) BA_1-GQD (**C**) BA_2-GQD (**D**) TPE_2-GQD

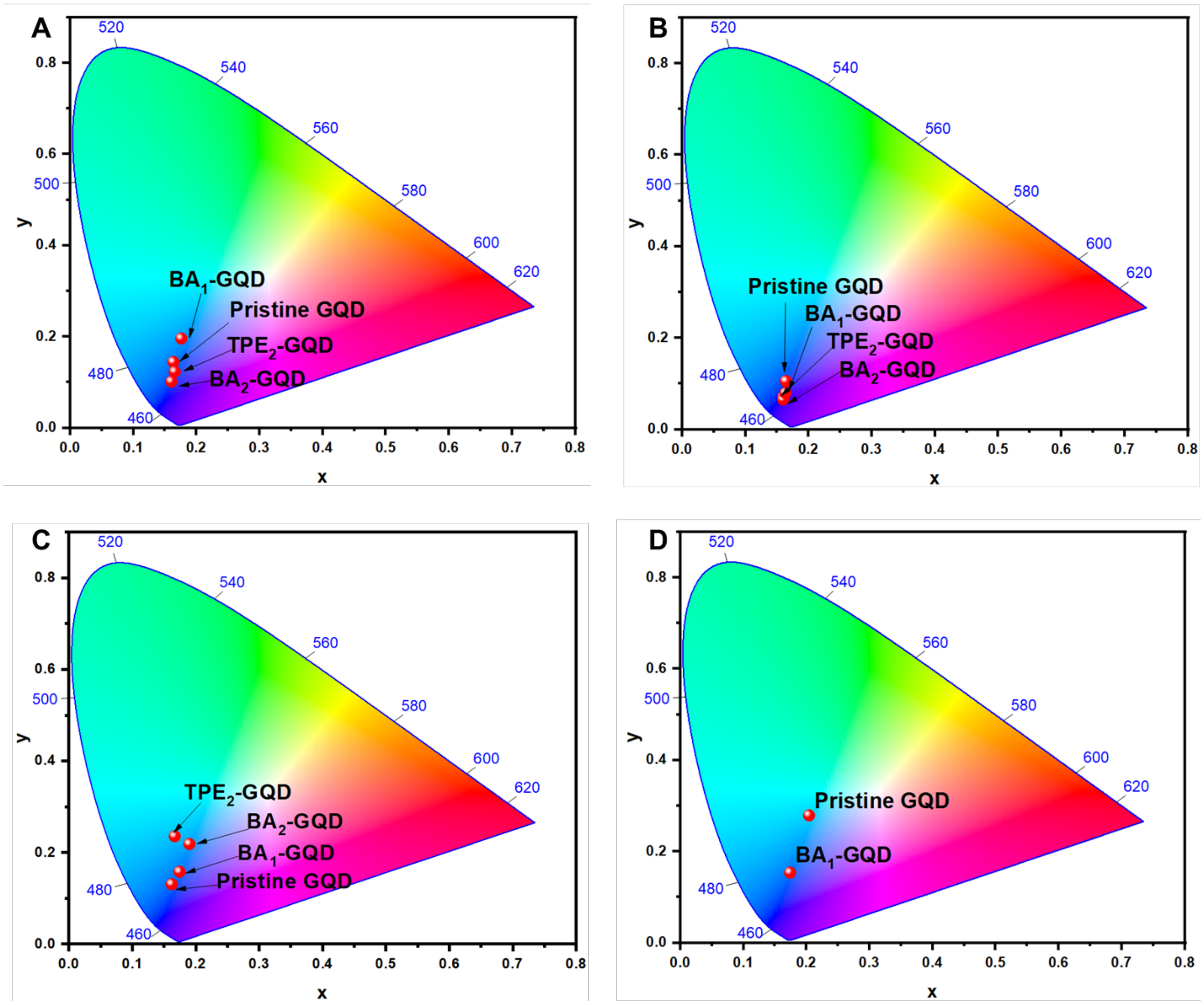

Fig. S8. Commission International de l'Eclairage (CIE) coordinates of GQDs for their prompt fluorescence in each state. (**A**) GQDs in solution (**B**) GQDs in f_{THF} = 90 (**C**) GQDs in powder (**D**) GQDs' afterglow.

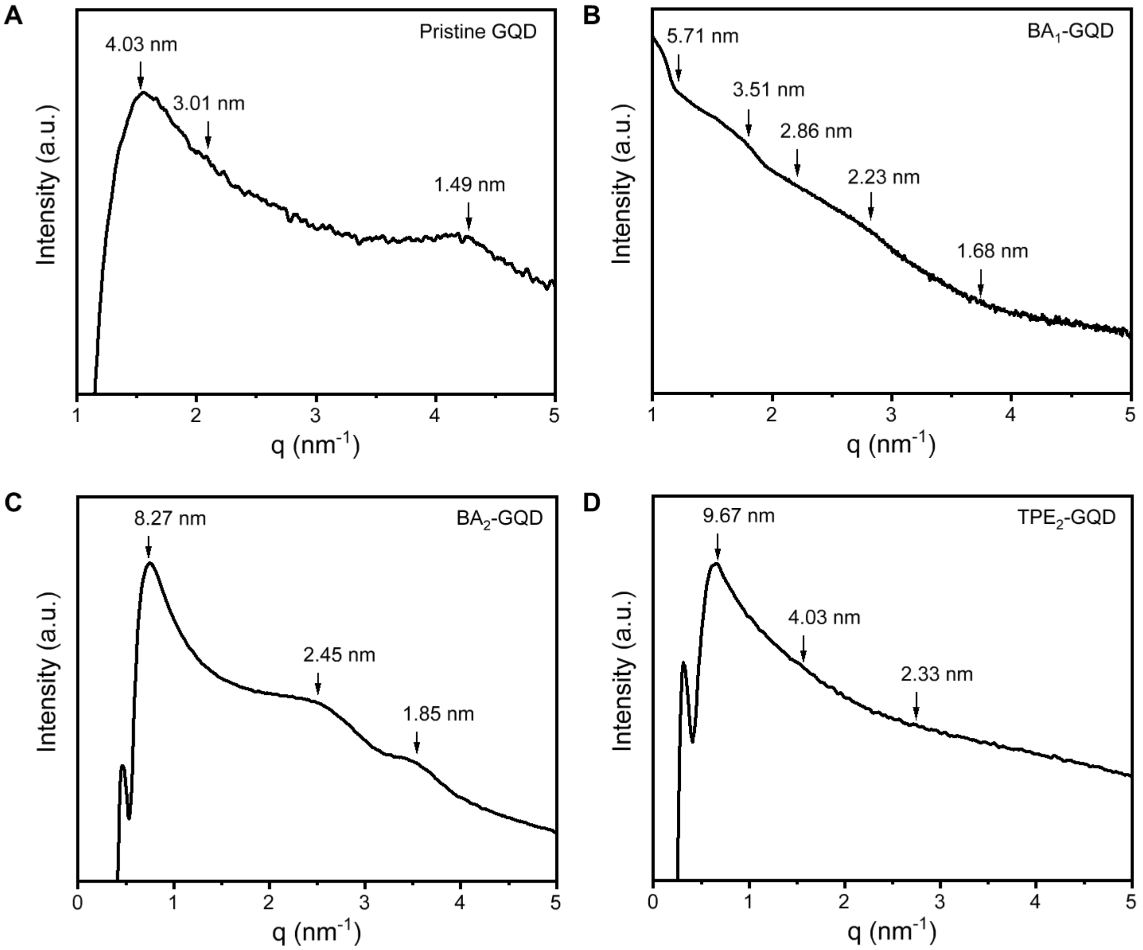

Fig. S9. Small angle x-ray scattering (SAXS) patterns of GQDs. (**A**) Pristine GQD (**B**) BA_1-GQD (**C**) BA_2-GQD (**D**) TPE_2-GQD

.

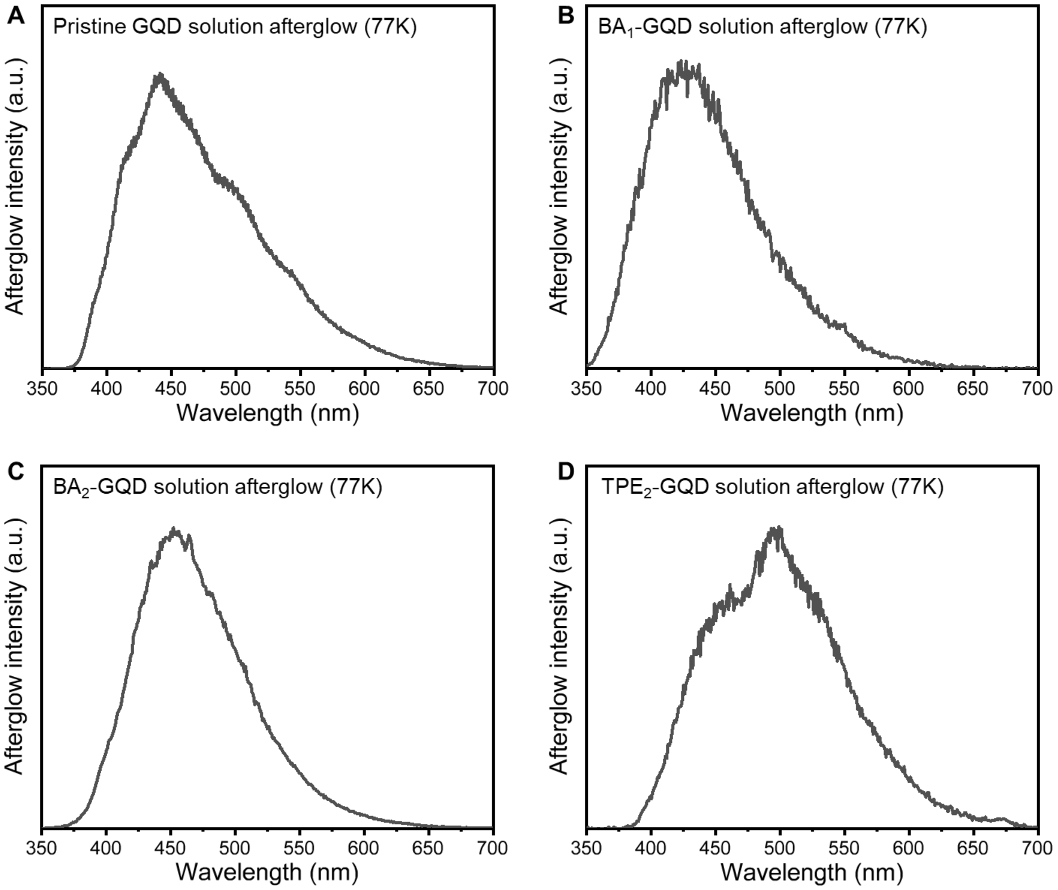

Fig. S10. Afterglow spectra of various GQDs in solution (f_{THF} = 0) measured at 77 K (λex = 285 nm). (**A**) Pristine GQDs (**B**) BA_1-GQDs (**C**) BA_2-GQDs (**D**) TPE_2-GQDs

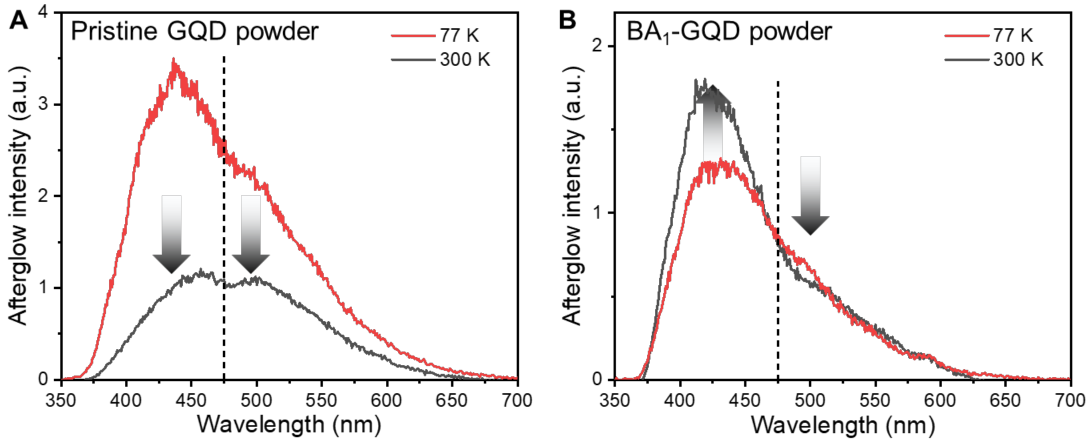

Fig. S11. Comparison on relative intensities of afterglow spectra of GQD powders at different temperatures at λ_{ex} = 285 nm. (**A**) Pristine GQDs (**B**) BA_1-GQDs

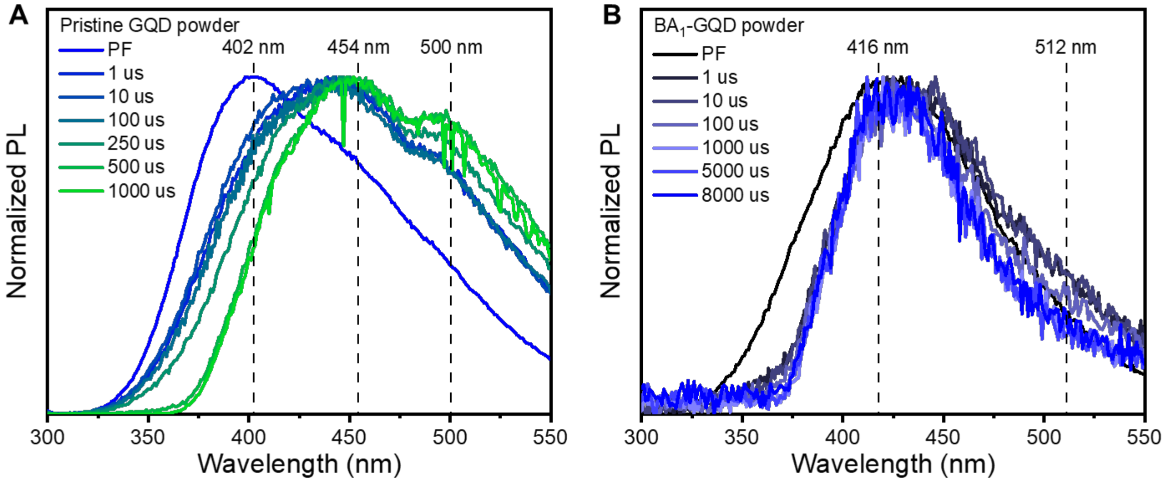

Fig. S12. Normalized time-resolved emission spectra (TRES) of afterglow-active GQD powders by varying gate delay time. (**A**) Pristine GQDs (**B**) BA_1-GQDs

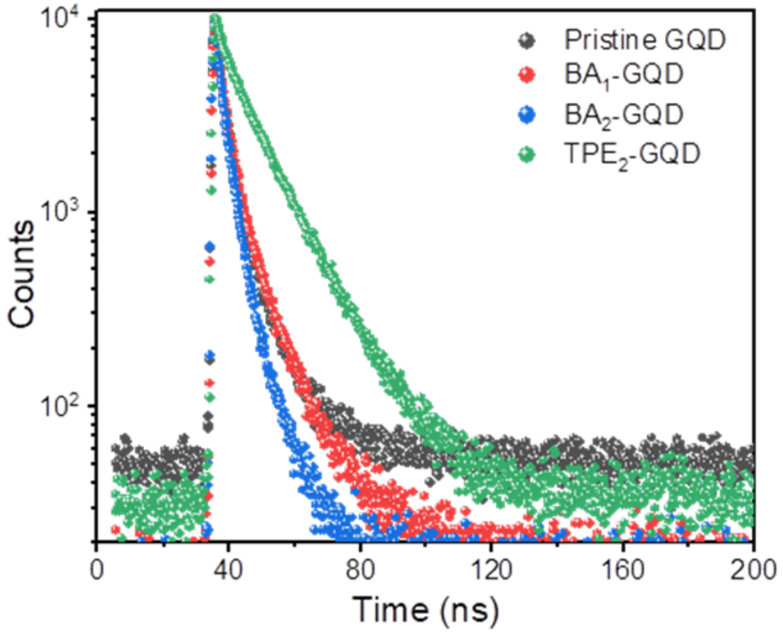

Fig. S13. Prompt fluorescence decay of various GQDs in solution (f_{THF} = 0)

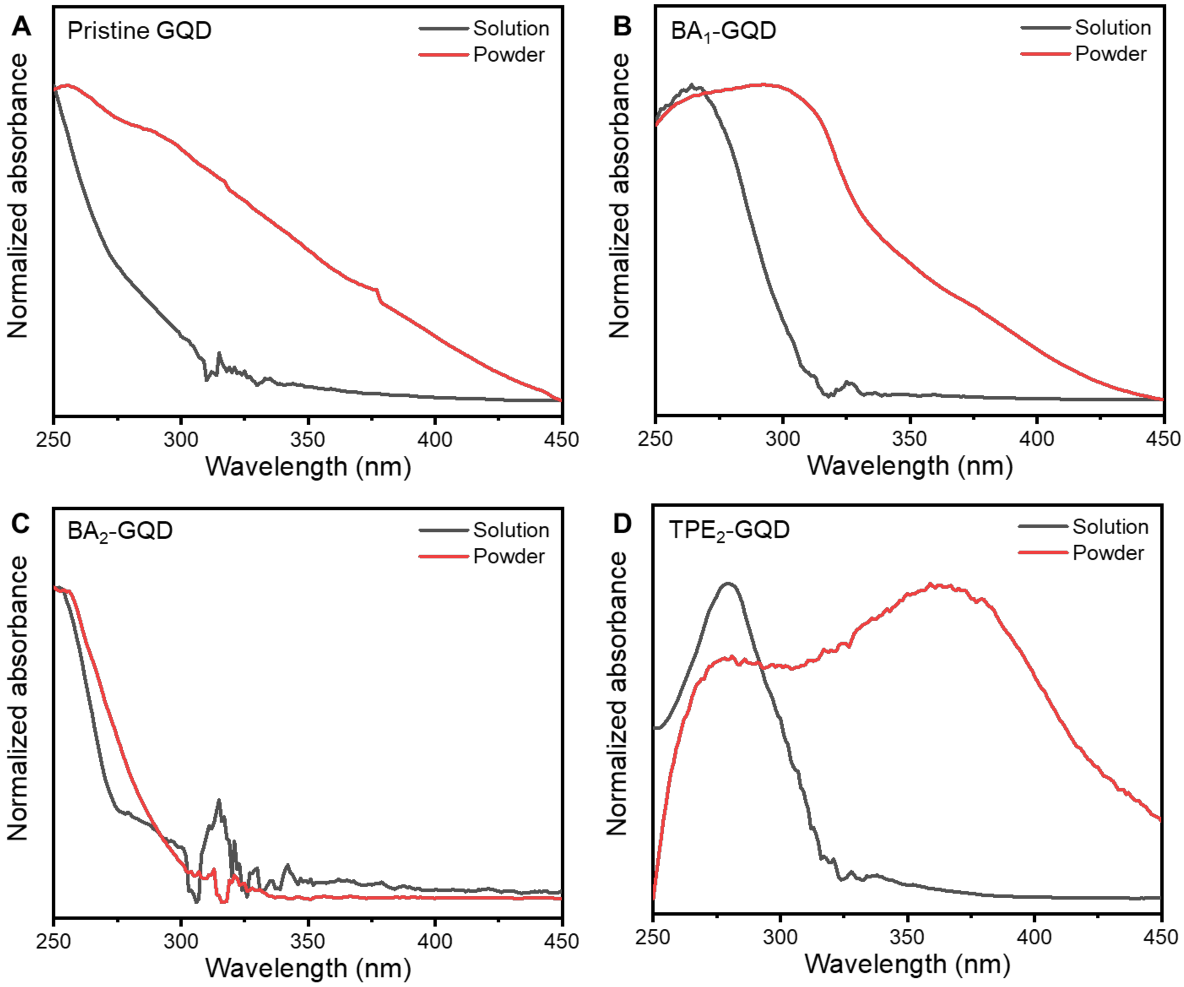

Fig. S14. Normalized absorption spectra of GQDs in solution at f_{THF}=0 (monomer) and powder. (**A**) Pristine GQD (**B**) BA_1-GQD (**C**) BA_2-GQD (**D**) TPE_2-GQD

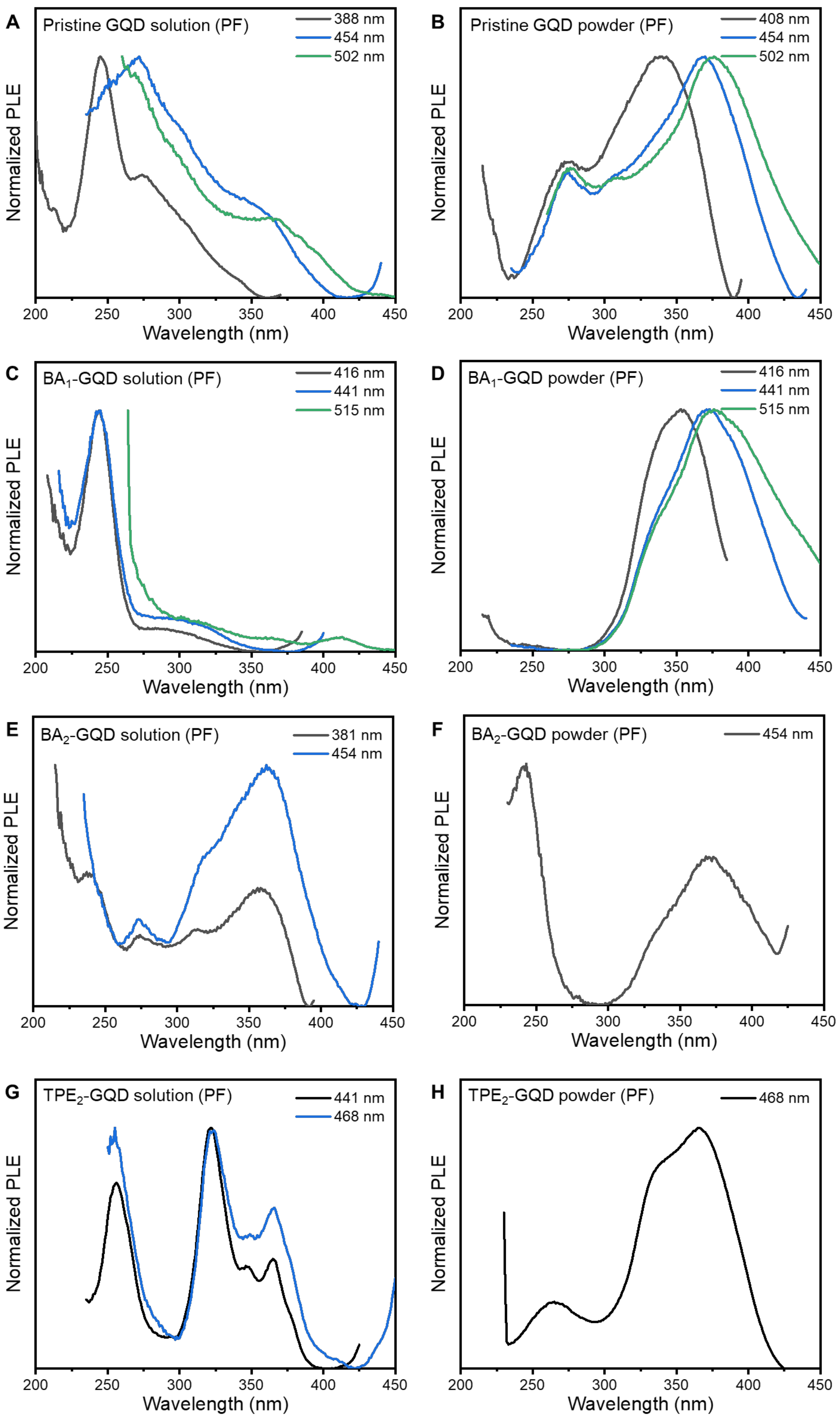
A
Pristine GQD solution (PF)
388 nm
454 nm
502 nm
B
Pristine GQD powder (PF)
408 nm
454 nm
502 nm
C
BA1-GQD solution (PF)
416 nm
441 nm
515 nm
D
BA1-GQD powder (PF)
416 nm
441 nm
515 nm
E
BA2-GQD solution (PF)
381 nm
454 nm
F
BA2-GQD powder (PF)
454 nm
G
TPE2-GQD solution (PF)
441 nm
468 nm
H
TPE2-GQD powder (PF)
468 nm
Normalized PLE
Wavelength (nm)
200
250
300
350
400
450

Fig. S15. Normalized photoluminescence excitation (PLE) spectra of GQDs PF. (**A**) Pristine GQD solution (**B**) Pristine GQD powder (**C**) BA_1-GQD solution (**D**) BA_1-GQD powder (**E**) BA_2-GQD solution (**F**) BA_2-GQD powder (**G**) TPE_2-GQD solution (**H**) TPE_2-GQD powder

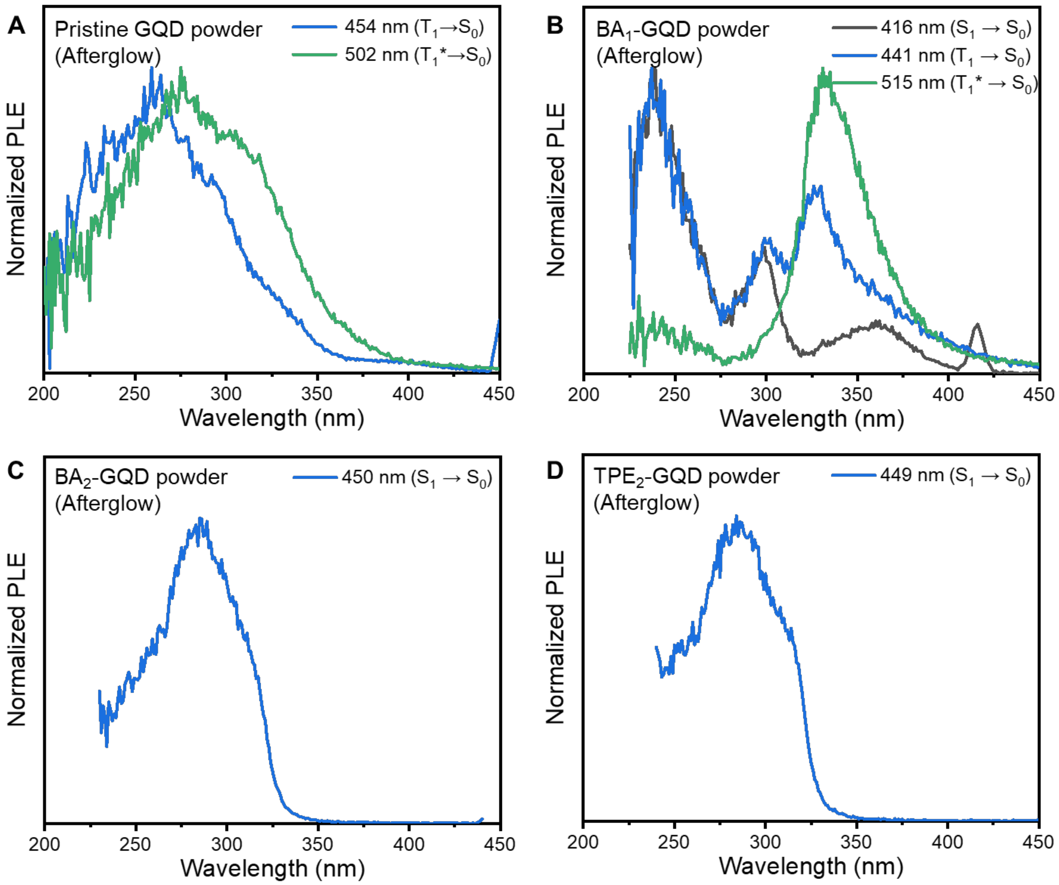

Fig. S16. Normalized PLE spectra of GQDs afterglow in powder at 300 K. (**A**) Pristine GQD (**B**) BA_1-GQD (**C**) BA_2-GQD (**D**) TPE_2-GQD

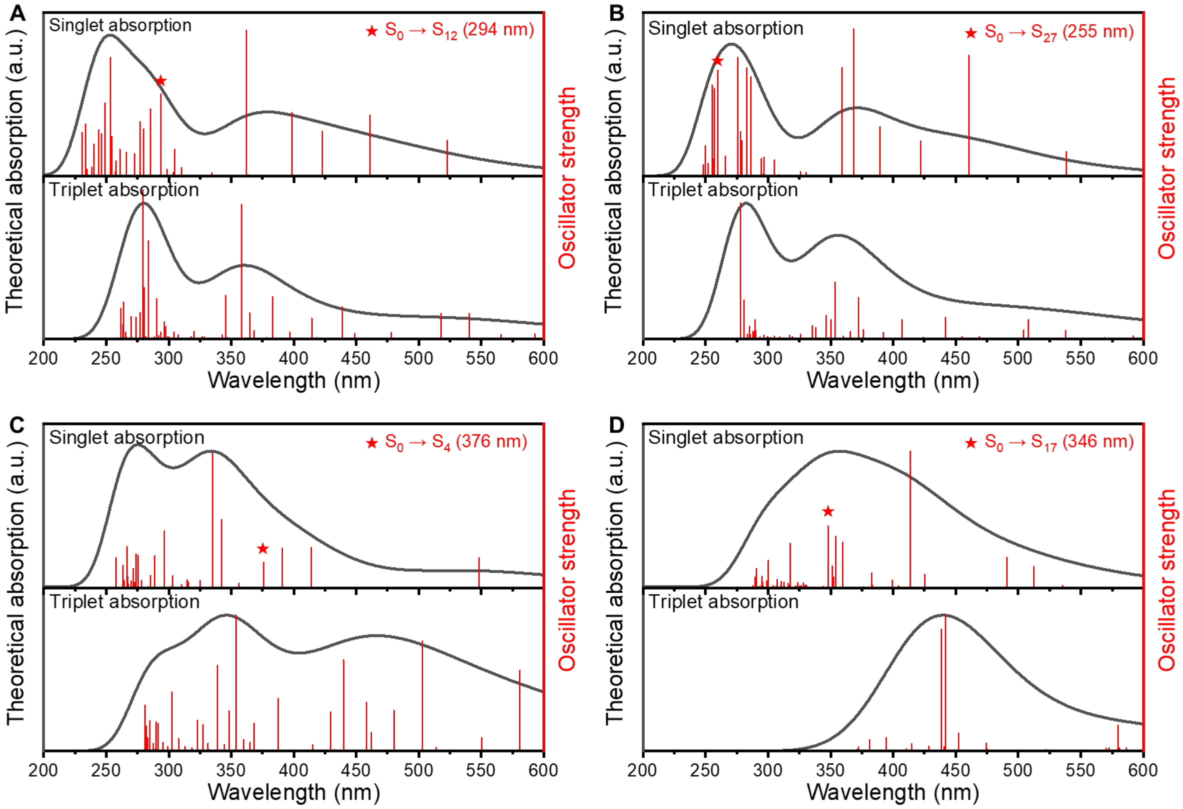

Fig. S17. Theoretical energy levels (absorption) and oscillator strengths of singlet and triplet states of various GQDs from the first lowest 50 energy levels. (**A**) Pristine GQDs (**B**) BA_1-GQDs (**C**) BA_2-GQDs (**D**) TPE_2-GQDs

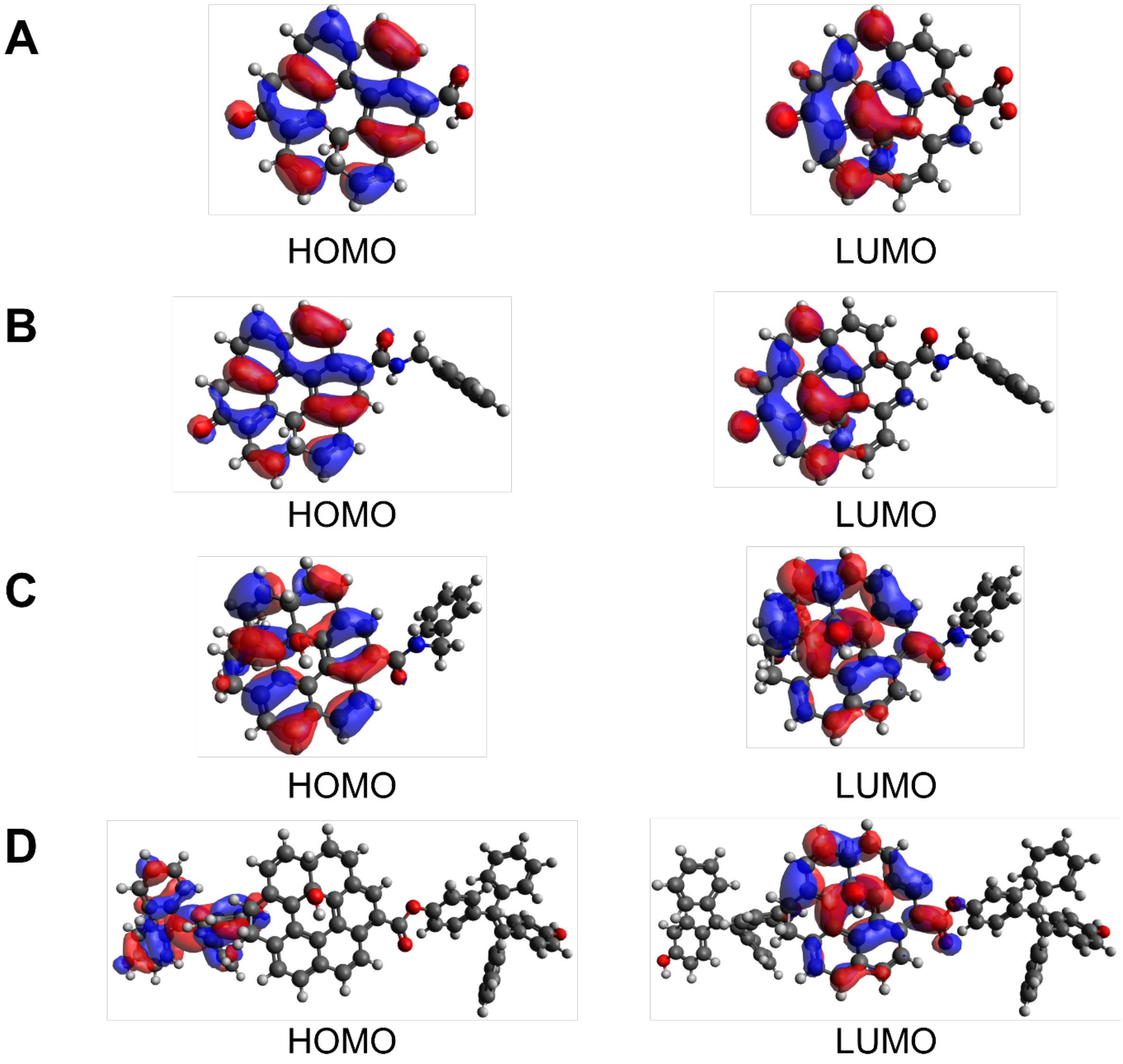

Fig. S18. The frontier orbitals of GQDs. (**A**) Pristine GQD (**B**) BA_1-GQD (**C**) BA_2-GQD (**D**) TPE_2-GQD

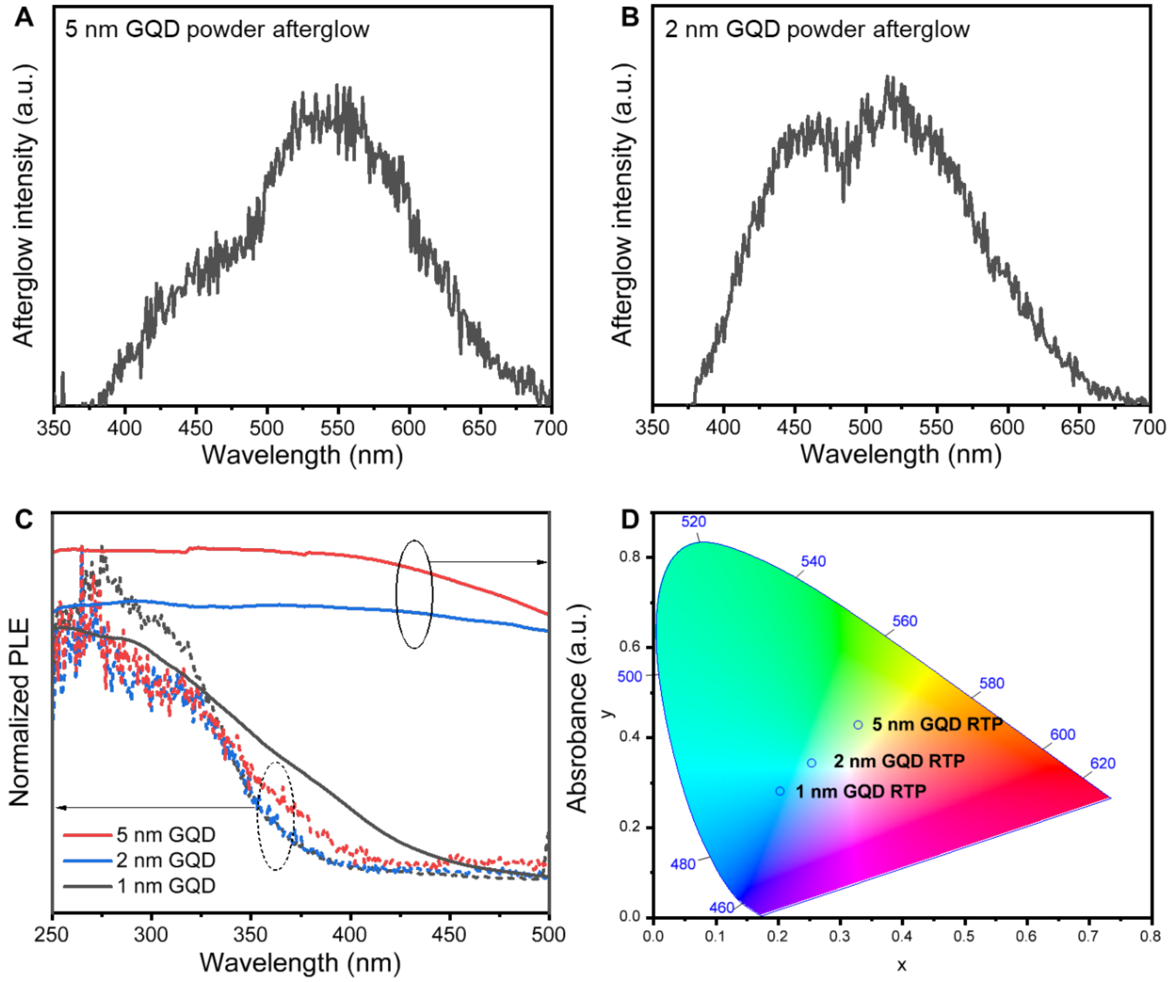

Fig. S19. RTP properties of 5 nm and 2 nm GQD powders. (**A**) Afterglow spectrum of 5 nm GQD powders. (**B**) Afterglow spectrum of 2 nm GQD powder. (**C**) Relative absorption spectra and normalized RTP PLE spectra of GQDs with sizes. (D) Commission International de I'Eclairage (CIE) coordinates of GQD RTP with sizes.

Supplementary text for fig. S19

Fig. S19A shows that the RTP for 5 nm GQDs peaked at 550 nm, which appears to be associated with intermolecular CT interactions. The 2 nm GQDs showed RTP characteristic peaks at 467 nm, and 516 nm, from monomer and powder, respectively, showed similar dual emission behaviors to the 1 nm GQDs (fig. S19B). Both their RTP from monomers and aggregates showed redshifted tendencies as the size of GQDs increased from 1 nm to 5 nm. It is noteworthy for 5 nm and 2 nm GQDs that due to the strong intermolecular CT throughout the visible ranges (fig. S19C),

RTP from intermolecular CT interaction is more pronounced for both GQDs, especially on 5 nm GQDs prominently. These phenomena can also be analyzed with PLE spectra of RTP (fig. S19C), which can be identified with a larger proportion of intermolecular CT bands for 5 nm at wavelengths > 350 nm. According to fig. S19D, along with PF, RTP can also be tuned simply by changing the size of GQDs even without being embedded in any matrices. The 2 nm and 5 nm GQDs showed cyan and yellowish-green emission from RTP, which is redshifted from the sky-blue RTP of the 1 nm GQDs (Fig. S19D). This can also be evidence that size reduction of GQD can effectively suppress the intermolecular CT interactions.

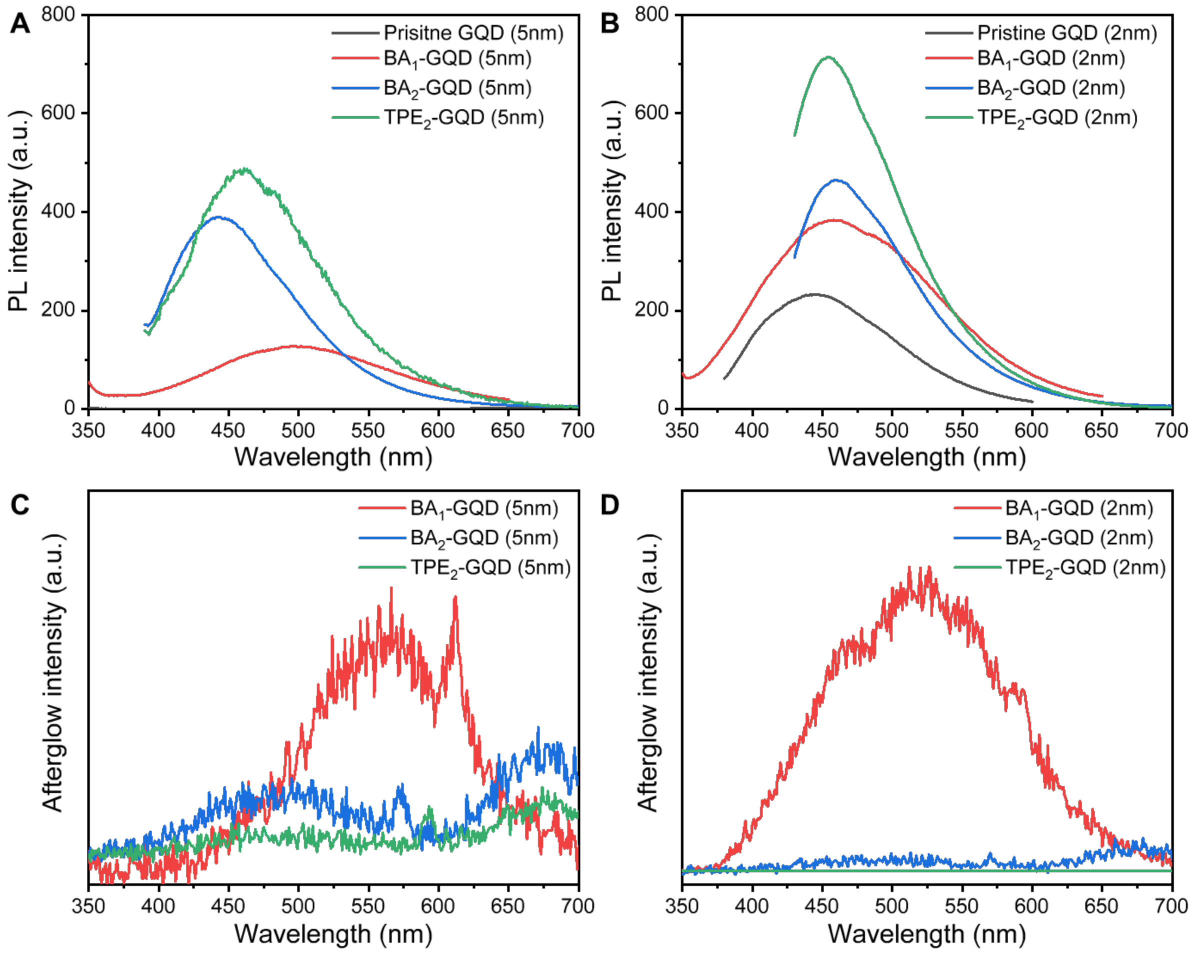

Fig. S20. PF and afterglow properties of as-functionalized 5 nm and 2 nm GQD powders. (**A**) 5 nm GQDs PF (**B**) 2 nm GQDs PF (**C**) 5 nm GQDs afterglow (**D**) 2nm GQDs afterglow

<u>Supplementary text for fig. S20</u>

Fig. S20 shows that the $\Phi_{F,powder}$ of 5 nm GQDs and 2 nm GQDs tend to increase in a similar manner as in the 1 nm GQDs. Each GQD exhibited $\Phi_{F,powder}$ of 0.5 % (pristine GQDs (5 nm)), 1.3 % (BA_1-GQDs (5 nm)), 4.0 % (BA_2-GQDs (5 nm)), 5.5 % (TPE_2-GQDs (5 nm)) for 5 nm GQDs and 1.2 % (pristine GQDs (2 nm)), 4.8 % (BA_1-GQDs (2 nm)), 6.2 % (BA_2-GQDs (2 nm)), 8.5 % (TPE_2-GQDs (2 nm)) for 5 nm GQDs. For fig. S20A, from BA_1-GQDs (5 nm), BA2-GQDs (5 nm) to TPE_2-GQDs (5 nm), however, their PL peaks correspond to 505 nm, 458 nm, 464

nm, respectively (fig. S20A). Strongly redshifted PL for BA_1-GQDs (5 nm) is probably due to the in-plane steric hindrances of BAs at the edges are insufficient for 5 nm GQDs to suppress intermolecular interactions. PL peaks of 2 nm GQDs powder followed analogous tendencies, 458 nm (BA_1-GQDs (2 nm)), 461 nm (BA_2-GQDs (2 nm)), and 462 nm (TPE_2-GQDs (2 nm)), meaning that each functionalization starts to suppress the π-π interactions more effectively than 5 nm cases (fig. S20B).

Afterglow properties of 5 nm and 2 nm GQDs are also investigated (fig. S20C-S20D). No afterglows for BA_2-GQDs and TPE_2-GQDs are observed for both sizes, as can be seen from 1 nm GQDs. This demonstrates that substitution of C=O to suppress the intramolecular CT is also valid regardless of their sizes. Although dim, BA_1-GQDs for both sizes exhibited afterglow, interestingly, unlike 1 nm case, RTP is more dominant than TADF, which is evidenced by higher intensity of RTP peak at 567 nm (5 nm), 525 nm (2 nm) than that of TADF at 524 nm (5 nm), 477 nm (2 nm) (fig. S20C-S20D). This may be attributed that multimer states dominates over monomer states, therefore aggregation-induced ISC affect stronger than those of 1 nm BA_1-GQDs. Therefore, it can be deduced that for larger GQDs, amide-functionalization with larger rotor molecules than BA are required to realize TADF from isolated monomers.

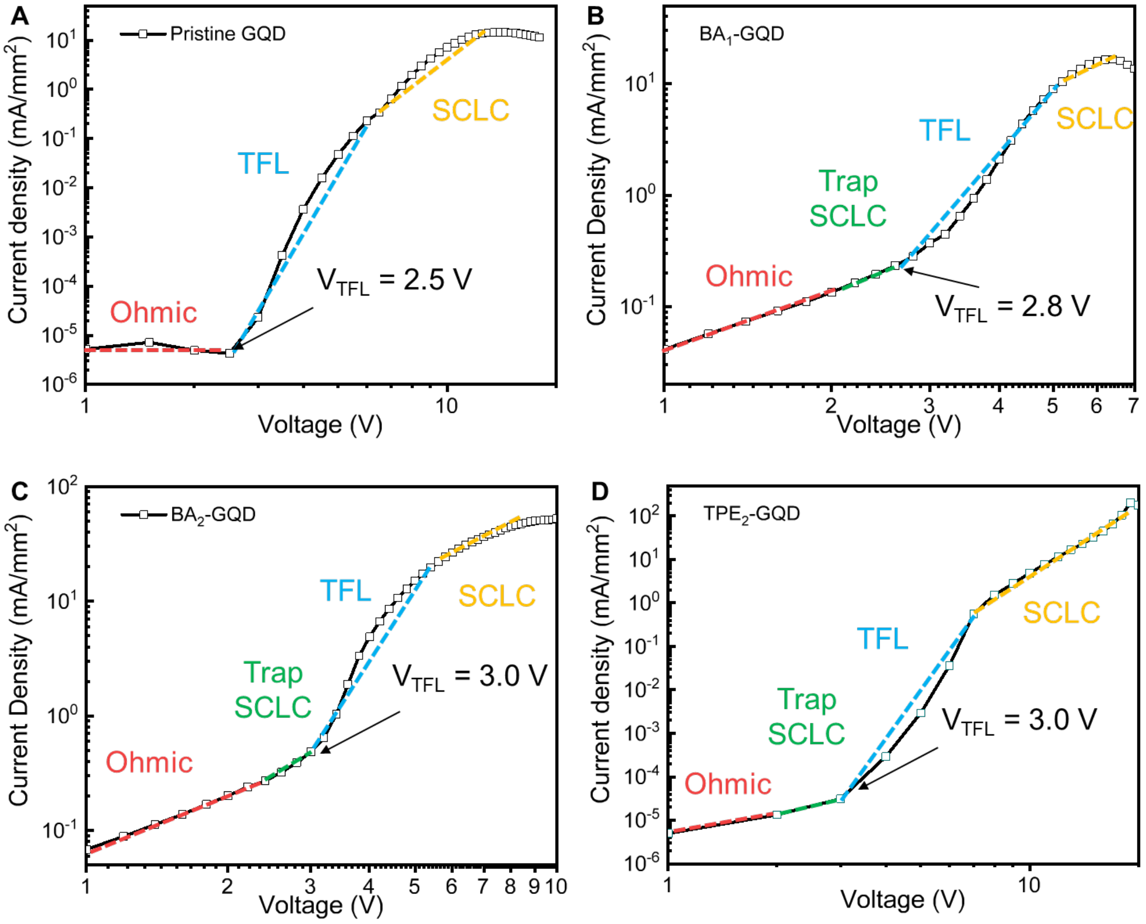

Fig. S21. Space charge limited current (SCLC) behavior of ITO/PEDOT:PSS/TFB/GQD /LiF/Al device. (**A**) Pristine GQD (**B**) BA_1-GQD (**C**) BA_2-GQD (**D**) TPE_2-GQD

Supplementary text for fig. S21

As demonstrated in fig. S21, most GQDs had four distinct charge transport regions: the ohmic, trap SCLC, TFL, and SCLC region. These regions are distinguished by their slopes, which are 1, 2, n>2 and 2 correspondingly. For pristine GQD (Fig. S21A), interestingly, trap SCLC region is not observed and directly moves to TFL region, implying the minimal influence of inherent traps in GQDs. Plus, V_{TFL} is approximately 2.5 V for pristine GQD, which is the lowest compared with other ligand-engineered GQDs, meaning that n_t is lowest, according to the Supplementary Equation (6). On the other hand, from fig. S21B - S21D, V_{TFL} of BA_1-GQD, BA_2-GQD, and TPE_2-

GQD is 2.8 V, 3.0 V, and 3.0 V, respectively, which still requires more potential for charges to hop between each GQD because the large BA and TPE-DOH ligands prevent charge injection. The high degree of π-π stacking between GQDs facilitates the intermolecular charge transport, making hopping easier to lower n_t and V_{TFL}. This explained that hopping is facilitated by π-electron overlaps between GQDs, in other words, introduction of bulkier ligands hinders charge transport from GQD to GQD.

However, J_{SCLC} exhibited opposite tendencies. TPE_2-GQD (Fig. S21D) showed the largest J_{SCLC} of 202.49 mA/mm^2 at V = 19 V, in contrast, J_{SCLC} of pristine GQD (Fig. S21A) is lower at its maxima, which is as small as 14.53 mA/mm^2 at V = 14 V. BA_1-GQD (Fig. S21B) and BA_2-GQD (Fig. S21C) showed J_{SCLC} of 16.54 mA/mm^2 at V = 6.4 V and 52.52 mA/mm^2 at V = 10 V. From Supplementary Equation (7), mobility (μ) of BA_1-GQD, BA_2-GQD and TPE_2-GQD is enhanced 5.44-, 7.08- and 7.57-fold than that of pristine GQD, also indicates that corresponding conductivity is also increased in a same manner. These aspects may be attributed to lowered built-in potential between hole transport layer (HTL) and ligand-engineered GQDs due to stabilized HOMO level compared to that of pristine GQD (Fig. 5A) in conjunction with charge delocalization ability stemming from the abundance of additional π-electrons provided by BA and TPE-DOH ligands compared to that of pristine GQDs.

Table S1. Properties of GQDs by size. Oxygen contents (%) obtained by XPS surveys and photoluminescence properties of GQDs with different sizes in aqueous solution and powder at their maximum PL intensity.

	**Oxygen contents (%)**	**Fluorescence**		**Quantum yield**	
		$\lambda_{em, soln}$ (nm)	$\lambda_{em, powder}$ (nm)	$\Phi_{F, soln}$ (%)	$\Phi_{F, powder}$ (%)
**5nm GQD**	26.12	407	–	4.5	0.5
**2nm GQD**	36.92	400	456	5.7	1.2
**1nm GQD**	42.34	388	450	6.8	2.5

Table S2. Size-dependent functional group distributions of GQDs. Binding energies and their relative atomic ratio of chemical bonds of GQDs with different sizes revealed by C1s XPS peaks.

	**Bond type**	**Binding energy (eV)**	**Atomic ratio (%)**
5 nm GQD	C=C	284.5	82.03
	C-OH	285.9	6.722
	C=O	287.8	5.756
	C=OOH	288.2	5.496
2 nm GQD	C=C	284.8	77.59
	C-OH	286.7	3.884
	C=O	287.6	10.76
	C=OOH	288.7	7.762
1 nm GQD (Pristine GQD)	C=C	284.5	62.71
	C-OH	285.9	9.573
	C=O	287.8	22.02
	C=OOH	289.2	5.697

Table S3. PL properties of GQDs in various states.

	Fluorescence						Quantum yield			
	$\lambda_{em, soln}$* (nm)	$\Delta\lambda_{soln}$* (nm)	$\lambda_{em, agg}$† (nm)	$\Delta\lambda_{agg}$† (nm)	$\lambda_{em, powder}$ (nm)	$\Delta\lambda_{powder}$ (nm)	$\Phi_{F, soln}$* (%)	$\Phi_{F, agg}$† (%)	$\Phi_{F, powder}$ (%)	α_{AIE}
**Pristine GQD**	388	98	377	76	450	125	6.8	1.0	2.5	0.36
**BA_1-GQD**	416	101	386, 404	78	441	104	3.2	8.5	7.9	2.47
**BA_2-GQD**	381	87	389, 404	74	454	128	5.1	15.6	15.6	3.05
**TPE_2-GQD**	414	100	388, 404	58	469	92	4.4	17.2	16.8	3.82

*Measured in f_{THF} = 0
†Measured in f_{THF} = 90

Table S4. GQD-to-GQD intermolecular distance derived from SAXS pattern of the various GQD powders.

	q_1^{max} (nm^{-1})	L_1 (nm)	q_2^{max} (nm^{-1})	L_2 (nm)	q_3^{max} (nm^{-1})	L_3 (nm)	q_4^{max} (nm^{-1})	L_4 (nm)	q_5^{max} (nm^{-1})	L_5 (nm)
**Pristine GQD**	4.21	1.49	2.09	3.01	1.56	4.03	-	-	-	-
**BA_1-GQD**	3.74	1.68	2.81	2.23	2.21	2.86	1.79	3.51	1.17	5.71
**BA_2-GQD**	3.40	1.85	2.56	2.45	0.76	8.27	-	-	-	-
**TPE_2-GQD**	2.71	2.33	1.56	4.03	0.65	9.67	-	-	-	-

Table S5. Energy levels of various GQDs in monomer and powder.

	Monomer			Powder			
	S_1 (eV)	*T_1 (eV)	ΔE_{ST} (eV)	S_1 (eV)	T_1 (eV)	†T_1* [a] (eV)	ΔE_{ST} (eV)
**Pristine GQD**	3.20	2.81	0.39	3.04	2.73	2.47	0.31
**BA_1-GQD**	3.10	2.86	0.24	2.98	2.81	2.42	0.17
**BA_2-GQD**	3.18	2.71	0.46	2.76	N/D	N/D	-
**TPE_2-GQD**	2.79	2.48	0.31	2.64	N/D	N/D	-

*T_1: T_1 states for monomers are determined by phosphorescence peaks of solution (f_{THF}=0) measured at 77 K
†T_1*: Newly appeared phosphorescence peak in powder form compared to their solution counterparts

Table S6. Prompt fluorescence lifetimes (τ_{PF}) of various GQDs in solution (f_{THF}=0).

	τ_1 (ns)	A_1	τ_2 (ns)	A_2	τ_3 (ns)	A_3	<τ> (ns)	χ^2	k_r ($\times10^7 s^{-1}$)	k_{nr} ($\times10^7 s^{-1}$)
**Pristine GQD**	1.253	6555	4.016	4193	12.21	492.0	4.689	1.093	1.397	19.14
**BA_1-GQD**	0.985	5877	3.531	4401	10.59	1019	5.445	1.163	0.588	17.78
**BA_2-GQD**	0.891	5453	2.896	5360	7.777	707.1	3.659	1.116	1.394	25.94
**TPE_2-GQD**	2.331	3271	12.47	5530	-	-	11.46	1.291	0.384	8.340

Table S7. Prompt fluorescence lifetimes (τ_{PF}) of various GQDs in powder.

	τ_1 (ns)	A_1	τ_2 (ns)	A_2	τ_3 (ns)	A_3	<τ> (ns)	χ^2	k_r ($\times 10^7 s^{-1}$)	k_{nr} ($\times 10^7 s^{-1}$)
**Pristine GQD**	0.779	3296	3.366	1459	10.70	344.7	5.194	1.116	0.481	18.77
**BA_1-GQD**	1.524	6783	5.503	3207	18.24	438.3	7.189	1.385	1.099	12.81
**BA_2-GQD**	0.590	1.000	4.654	8301	-	-	4.654	0.925	3.352	18.14
**TPE_2-GQD**	0.747	5004	2.242	5012	-	-	1.869	1.197	8.989	44.52

Table S8. Delayed fluorescence and phosphorescence lifetimes of various GQDs in powder.

		τ_1 (ms)	A_1	τ_2 (ms)	A_2	τ_3 (ms)	A_3	τ_4 (ms)	A_4	$\langle\tau\rangle$ (ms)	χ^2
**DF**	**Pristine GQD**	0.631	9555	64.39	213.4	325.6	140.3	-	-	240.9	1.095
	**BA_1-GQD**	0.199	59798	3.144	2574	33.00	1003	157.8	731.4	114.8	1.088
**RTP**	**Pristine GQD**	0.574	9917	45.70	77.63	437.8	58.46	-	-	326.4	1.126
	**BA_1-GQD**	0.140	62030	2.639	1324	24.29	142.1	233.7	54.71	108.5	1.022

Table S9. Summary of the photophysical properties of reported GQDs and carbon dots.

Entry	Material	Synthesis	Matrix	Emission (Color, nm)	Emission type	Solid-state PLQY (%)	Ref.
	**Pristine GQD**	**Top-down (Graphite intercalation compounds)**	**None**	**B (450 nm)**<br>**G (500 nm)**	**Dual-emissive**<br>**RTP**	**2.5**	**This work**
	**BA_1-GQD**			**B (441 nm)**	**TADF**	**8.5**	
	**BA_2-GQD**			**B (454 nm)**	**Fluorescence**	**15.6**	
	**TPE_2-GQD**			**B (469 nm)**	**Fluorescence**	**16.8**	
1	GQDs-Agar	Top-down (Glucose Hydrothermal)	Agar	B (480 nm)	Fluorescence	12 in matrix	(*23*)
2	N-GQDs	Top-down (Hummers method)	Cellulose nanofiber-clay	B (450 nm)<br>G (525 nm)	Fluorescence	-	(*71*)
3	TPS/GQD	Top-down (Hummers method)	Thermoplastic starch	B (455 nm)	Fluorescence	8.25 in matrix	(*72*)
4	N-GQDs	Bottom-up (Citric acid monohydrate diethylenetriamine)	None	G (514 nm)	Fluorescence	10	(*73*)
5	GQD@ZIF-8	Bottom-up (Molecular fusion of 1,3,6-trinitropyrene and Na_2SO_3)	ZIF-8	Y (490 nm)	Fluorescence	26.1 in matrix	(*74*)
6	TA-CDs	Bottom-up (Trimellitic acid hydrothermal)	None	W (430 nm)<br>Y (580 nm)	Fluorescence<br>RTP	4.8	(*29*)
7	URTP CDs	Bottom-up (Ethanolamine and phosphoric acid)	None	B (450 nm)	RTP	3.53	(*75*)
8	CDs	Bottom-up (AEAPMS + citric acid)	None	R (650 nm)	Fluorescence	9.60	(*76*)
9	EDA-CDs	Bottom-up (Pyrolysis of ethylenediamine (EDA) in phosphoric acid)	None	B (450 nm)	RTP	14.01	(*77*)
10	CDs@AlPO-5<br>CDs@2D-AlPO<br>CDs@MgAPO-5	Bottom-up (Solvothermal)	AIPO-5<br>2D-AIPO<br>MgAPO-5	B (430 nm)<br>B (440 nm)<br>B (425 nm)	TADF<br>TADF<br>TADF	15.53 in matrix<br>52.14 in matrix<br>22.77 in matrix	(*78*)
11	CDs<br>CDs@MOF-1<br>CMOF-1	Bottom-up (p-phenylenediamine + ethylenediamine solvothermal)	None<br>MOF-1<br>MOF	G (518 nm)<br>R (600 nm)<br>B (470 nm)	Fluorescence<br>Fluorescence<br>Fluorescence	16.94<br>26.2 in matrix<br>44.83 in matrix	(*79*)

* RGBYW denotes each emission color (R:Red, G:Green, B:Blue, Y:Yellow, W:White)

Movie S1. Afterglow of pristine GQD and BA_1-GQD powders under irradiation with 315 nm UV lamp

REFERENCES AND NOTES

1. J. Kim, S. H. Song, Y. Jin, H. J. Park, H. Yoon, S. Jeon, S. W. Cho, Multiphoton luminescent graphene quantum dots for in vivo tracking of human adipose-derived stem cells. *Nanoscale* **8**, 8512–8519 (2016).

2. H. Yoon, M. Park, J. Kim, T. G. Novak, S. Lee, S. Jeon, Toward highly efficient luminescence in graphene quantum dots for optoelectronic applications. *Chem. Phys. Rev.* **2**, 031303 (2021).

3. S. H. Song, M.-H. Jang, J.-M. Jeong, H. Yoon, Y.-H. Cho, W.-I. Jeong, B.-H. Kim, S. Jeon, Primary hepatocyte imaging by multiphoton luminescent graphene quantum dots. *Chem. Commun.* **51**, 8041–8043 (2015).

4. M. Park, Y. Jeong, H. S. Kim, W. Lee, S.-H. Nam, S. Lee, H. Yoon, J. Kim, S. Yoo, S. Jeon, Quenching-resistant solid-state photoluminescence of graphene quantum dots: Reduction of π−π stacking by surface functionalization with POSS, PEG, and HDA. *Adv. Funct. Mater.* **31**, 2102741 (2021).

5. L. Wang, Y. Wang, T. Xu, H. Liao, C. Yao, Y. Liu, Z. Li, Z. Chen, D. Pan, L. Sun, M. Wu, Gram-scale synthesis of single-crystalline graphene quantum dots with superior optical properties. *Nat. Commun.* **5**, 5357 (2014).

6. S. Zhu, J. Zhang, S. Tang, C. Qiao, L. Wang, H. Wang, X. Liu, B. Li, Y. Li, W. Yu, X. Wang, H. Sun, B. Yang, Surface chemistry routes to modulate the photoluminescence of graphene quantum dots: From fluorescence mechanism to up-conversion bioimaging applications. *Adv. Funct. Mater.* **22**, 4732–4740 (2012).

7. S. J. Bradley, R. Kroon, G. Laufersky, M. Röding, R. V. Goreham, T. Gschneidtner, K. Schroeder, K. Moth-Poulsen, M. Andersson, T. Nann, Heterogeneity in the fluorescence of graphene and graphene oxide quantum dots. *Microchim. Acta* **184**, 871–878 (2017).

8. S. Ahirwar, S. Mallick, D. Bahadur, Electrochemical method to prepare graphene quantum dots and graphene oxide quantum dots. *ACS Omega* **2**, 8343–8353 (2017).

9. H. Yoon, H. S. Kim, J. Kim, M. Park, B. Kim, S. Lee, K. Kang, S. Yoo, S. Jeon, Blue graphene quantum dots with high color purity by controlling subdomain formation for light-emitting devices. *ACS Appl. Nano Mater.* **3**, 6469–6477 (2020).

10. S. H. Song, M.-H. Jang, J. Chung, S. H. Jin, B. H. Kim, S.-H. Hur, S. Yoo, Y.-H. Cho, S. Jeon, Highly efficient light-emitting diode of graphene quantum dots fabricated from graphite intercalation compounds. *Adv. Opt. Mater.* **2**, 1016–1023 (2014).

11. Z. Gan, H. Xu, Y. Fu, Photon reabsorption and nonradiative energy-transfer-induced quenching of blue photoluminescence from aggregated graphene quantum dots. *J. Phys. Chem. C* **120**, 29432–29438 (2016).

12. J. Kwon, S. H. Lee, K.-H. Park, D.-H. Seo, J. Lee, B.-S. Kong, K. Kang, S. Jeon, Simple preparation of high-quality graphene flakes without oxidation using potassium salts. *Small* **7**, 864–868 (2011).

13. K. H. Park, B. H. Kim, S. H. Song, J. Kwon, B. S. Kong, K. Kang, S. Jeon, Exfoliation of non-oxidized graphene flakes for scalable conductive film. *Nano Lett.* **12**, 2871–2876 (2012).

14. S. H. Song, K. H. Park, B. H. Kim, Y. W. Choi, G. H. Jun, D. J. Lee, B.-S. Kong, K.-W. Paik, S. Jeon, Enhanced thermal conductivity of epoxy-graphene composites by using non-oxidized graphene flakes with non-covalent functionalization. *Adv. Mater.* **25**, 732–737 (2013).

15. J. Kim, S. H. Song, H.-G. Im, G. Yoon, D. Lee, C. Choi, J. Kim, B.-S. Bae, K. Kang, S. Jeon, Moisture barrier composites made of non-oxidized graphene flakes. *Small* **11**, 3124–3129 (2015).

16. J. Kim, J. Kim, S. Song, S. Zhang, J. Cha, K. Kim, H. Yoon, Y. Jung, K.-W. Paik, S. Jeon, Strength dependence of epoxy composites on the average filler size of non-oxidized graphene flake. *Carbon* **113**, 379–386 (2017).

17. J. Kim, N. M. Han, J. Kim, J. Lee, J.-K. Kim, S. Jeon, Highly conductive and fracture-resistant epoxy composite based on non-oxidized graphene flake aerogel. *ACS Appl. Mater. Interfaces* **10**, 37507–37516 (2018).

18. H. Yoon, Y. H. Chang, S. H. Song, E.-S. Lee, S. H. Jin, C. Park, J. Lee, B. H. Kim, H. J. Kang, Y.-H. Kim, S. Jeon, Intrinsic photoluminescence emission from subdomained graphene quantum dots. *Adv. Mater.* **28**, 5255–5261 (2016).

19. M. Park, H. S. Kim, H. Yoon, J. Kim, S. Lee, S. Yoo, S. Jeon, Controllable singlet-triplet energy splitting of graphene quantum dots through oxidation: From phosphorescence to TADF. *Adv. Mater.* **32**, 2000936 (2020).

20. M. Park, H. Yoon, J. Lee, J. Kim, J. Lee, S.-E. Lee, S. Yoo, S. Jeon, Efficient solid-state photoluminescence of graphene quantum dots embedded in boron oxynitride for AC-electroluminescent device. *Adv. Mater.* **30**, 1802951 (2018).

21. A. Kovalchuk, K. Huang, C. Xiang, A. A. Martí, J. M. Tour, Luminescent polymer composite films containing coal-derived graphene quantum dots. *ACS Appl. Mater. Interfaces* **7**, 26063–26068 (2015).

22. J. Chen, Z. Long, S. Wang, Y. Meng, G. Zhang, S. Nie, Biodegradable blends of graphene quantum dots and thermoplastic starch with solid-state photoluminescent and conductive properties. *Int. J. Biol. Macromol.* **139**, 367–376 (2019).

23. C. M. Luk, L. B. Tang, W. F. Zhang, S. F. Yu, K. S. Teng, S. P. Lau, An efficient and stable fluorescent graphene quantum dot–agar composite as a converting material in white light emitting diodes. *J. Mater. Chem.* **22**, 22378–22381 (2012).

24. J. Kyu Kim, S. Bae, Y. Yi, M. Jin Park, S. Jin Kim, N. Myoung, C.-L. Lee, B. Hee Hong, J. Hyeok Park, Origin of white electroluminescence in graphene quantum dots embedded host/guest polymer light emitting diodes. *Sci. Rep.* **5**, 11032 (2015).

25. T. Scharl, A. Ferrer-Ruiz, A. Saura-Sanmartín, L. Rodríguez-Pérez, M. Á. Herranz, N. Martín, D. M. Guldi, Charge transfer in graphene quantum dots coupled with tetrathiafulvalenes. *Chem. Commun.* **55**, 3223–3226 (2019).

26. J. Luo, Z. Xie, J. W. Lam, L. Cheng, H. Chen, C. Qiu, H. S. Kwok, X. Zhan, Y. Liu, D. Zhu, B. Z. Tang, Aggregation-induced emission of 1-methyl-1,2,3,4,5-pentaphenylsilole. *Chem. Commun.* **2001**, 1740–1741 (2001).

27. B.-G. Kim, E. J. Jeong, H. J. Park, D. Bilby, L. J. Guo, J. Kim, Effect of polymer aggregation on the open circuit voltage in organic photovoltaic cells: Aggregation-induced conjugated polymer gel and its application for preventing open circuit voltage drop. *ACS Appl. Mater. Interfaces* **3**, 674–680 (2011).

28. G. Liang, J. W. Y. Lam, W. Qin, J. Li, N. Xie, B. Z. Tang, Molecular luminogens based on restriction of intramolecular motions through host-guest inclusion for cell imaging. *Chem. Commun.* **50**, 1725–1727 (2014).

29. K. Jiang, X. Gao, X. Feng, Y. Wang, Z. Li, H. Lin, Carbon dots with dual-emissive, robust, and aggregation-induced room-temperature phosphorescence characteristics. *Angew. Chem. Int. Ed.* **59**, 1263–1269 (2020).

30. O. Bolton, K. Lee, H.-J. Kim, K. Y. Lin, J. Kim, Activating efficient phosphorescence from purely organic materials by crystal design. *Nat. Chem.* **3**, 205–210 (2011).

31. M. S. Kwon, Y. Yu, C. Coburn, A. W. Phillips, K. Chung, A. Shanker, J. Jung, G. Kim, K. Pipe, S. R. Forrest, J. H. Youk, J. Gierschner, J. Kim, Suppressing molecular motions for enhanced room-temperature phosphorescence of metal-free organic materials. *Nat. Commun.* **6**, 8947 (2015).

32. Q. Zhang, D. Tsang, H. Kuwabara, Y. Hatae, B. Li, T. Takahashi, S. Y. Lee, T. Yasuda, C. Adachi, Nearly 100% internal quantum efficiency in undoped electroluminescent devices employing pure organic emitters. *Adv. Mater.* **27**, 2096–2100 (2015).

33. J. Wang, J. Zhang, C. Jiang, C. Yao, X. Xi, Effective design strategy for aggregation-induced emission and thermally activated delayed fluorescence emitters achieving 18% external quantum efficiency pure-blue OLEDs with extremely low roll-off. *ACS Appl. Mater. Interfaces* **13**, 57713–57724 (2021).

34. M. Kim, S.-J. Yoon, S. H. Han, R. Ansari, J. Kieffer, J. Y. Lee, J. Kim, Molecular design approach managing molecular orbital superposition for high efficiency without color shift in thermally activated delayed fluorescent organic light-emitting diodes. *Chem. A Eur. J.* **25**, 1829–1834 (2019).

35. T. Wang, X. Su, X. Zhang, X. Nie, L. Huang, X. Zhang, X. Sun, Y. Luo, G. Zhang, Aggregation-induced dual-phosphorescence from organic molecules for nondoped light-emitting diodes. *Adv. Mater.* **31**, 1904273 (2019).

36. H. Li, Z. Chi, B. Xu, X. Zhang, X. Li, S. Liu, Y. Zhang, J. Xu, Aggregation-induced emission enhancement compounds containing triphenylamine-anthrylenevinylene and tetraphenylethene moieties. *J. Mater. Chem.* **21**, 3760–3767 (2011).

37. C. A. Hunter, J. K. M. Sanders, The nature of .pi.-.pi. interactions. *J. Am. Chem. Soc.* **112**, 5525–5534 (1990).

38. G. Rajender, P. K. Giri, Formation mechanism of graphene quantum dots and their edge state conversion probed by photoluminescence and Raman spectroscopy. *J. Mater. Chem. C* **4**, 10852–10865 (2016).

39. M. W. Smith, I. Dallmeyer, T. J. Johnson, C. S. Brauer, J.-S. McEwen, J. F. Espinal, M. Garcia-Perez, Structural analysis of char by Raman spectroscopy: Improving band assignments through computational calculations from first principles. *Carbon* **100**, 678–692 (2016).

40. H.-H. Cho, H. Yang, D. J. Kang, B. J. Kim, Surface engineering of graphene quantum dots and their applications as efficient surfactants. *ACS Appl. Mater. Interfaces* **7**, 8615–8621 (2015).

41. S. Sato, T. Sakamoto, E. Miyazawa, Y. Kikugawa, One-pot reductive amination of aldehydes and ketones with α-picoline-borane in methanol, in water, and in neat conditions. *Tetrahedron* **60**, 7899–7906 (2004).

42. A. Jordan, K. D. Whymark, J. Sydenham, H. F. Sneddon, A solvent-reagent selection guide for Steglich-type esterification of carboxylic acids. *Green Chem.* **23**, 6405–6413 (2021).

43. S. S. Mochalov, A. N. Fedotov, E. V. Trofimova, N. S. Zefirov, Direct synthesis of ethers from aldehydes and ketones. One-pot reductive etherification of benzaldehydes, alkyl aryl ketones, and benzophenones. *Russ. J. Org. Chem.* **52**, 503–512 (2016).

44. Y. Niko, Y. Hiroshige, S. Kawauchi, G. Konishi, Additional insights into luminescence process of polycyclic aromatic hydrocarbons with carbonyl groups: Photophysical properties of secondary *N*-alkyl and tertiary *N*,*N*-dialkyl carboxamides of naphthalene, anthracene, and pyrene. *J. Org. Chem.* **77**, 3986–3996 (2012).

45. J. F. Engels, J. Roose, D. S. Zhai, K. M. Yip, M. S. Lee, B. Z. Tang, R. Renneberg, Aggregation-induced emissive nanoparticles for fluorescence signaling in a low cost paper-based immunoassay. *Colloids Surf. B Biointerfaces* **143**, 440–446 (2016).

46. L. G. Franca, Y. Long, C. Li, A. Danos, A. Monkman, The critical role of nπ* states in the photophysics and thermally activated delayed fluorescence of spiro acridine-anthracenone. *J. Phys. Chem. Lett.* **12**, 1490–1500 (2021).

47. T. Liu, C. Tonnelé, S. Zhao, L. Rondin, C. Elias, D. Medina-Lopez, H. Okuno, A. Narita, Y. Chassagneux, C. Voisin, S. Campidelli, D. Beljonne, J.-S. Lauret, Vibronic effect and influence of aggregation on the photophysics of graphene quantum dots. *Nanoscale* **14**, 3826–3833 (2022).

48. R. Hu, E. Lager, A. Aguilar-Aguilar, J. Liu, J. W. Y. Lam, H. H. Y. Sung, I. D. Williams, Y. Zhong, K. S. Wong, E. Peña-Cabrera, B. Z. Tang, Twisted intramolecular charge transfer and aggregation-induced emission of BODIPY derivatives. *J. Phys. Chem. C* **113**, 15845–15853 (2009).

49. J. Wang, X. Gu, P. Zhang, X. Huang, X. Zheng, M. Chen, H. Feng, R. T. K. Kwok, J. W. Y. Lam, B. Z. Tang, Ionization and anion-π^+ interaction: A new strategy for structural design of aggregation-induced emission luminogens. *J. Am. Chem. Soc.* **139**, 16974–16979 (2017).

50. W. Cai, D. Piner Richard, J. Stadermann Frank, S. Park, A. Shaibat Medhat, Y. Ishii, D. Yang, A. Velamakanni, J. An Sung, M. Stoller, J. An, D. Chen, S. Ruoff Rodney, Synthesis and solid-state NMR structural characterization of ^{13}C-labeled graphite oxide. *Science* **321**, 1815–1817 (2008).

51. R. Sekiya, Y. Uemura, H. Murakami, T. Haino, White-light-emitting edge-functionalized graphene quantum dots. *Angew. Chem. Int. Ed.* **53**, 5619–5623 (2014).

52. C. Balachandra, N. K. Sharma, Direct/reversible amidation of troponyl alkylglycinates via cationic troponyl lactones and mechanistic insights. *ACS Omega* **3**, 997–1013 (2018).

53. T. Phan-Xuan, E. Bogdanova, A. Millqvist Fureby, J. Fransson, A. E. Terry, V. Kocherbitov, Hydration-induced structural changes in the solid state of protein: A SAXS/WAXS study on lysozyme. *Mol. Pharm.* **17**, 3246–3258 (2020).

54. K. Liu, D. Chen, A. Marcozzi, L. Zheng, J. Su, D. Pesce, W. Zajaczkowski, A. Kolbe, W. Pisula, K. Müllen, N. A. Clark, A. Herrmann, Thermotropic liquid crystals from biomacromolecules. *Proc. Natl. Acad. Sci. U.S.A.* **111**, 18596–18600 (2014).

55. L. Bai, N. Xue, Y. Zhao, X. Wang, C. Lu, W. Shi, Dual-mode emission of single-layered graphene quantum dots in confined nanospace: Anti-counterfeiting and sensor applications. *Nano Res.* **11**, 2034–2045 (2018).

56. X. Wang, Y. Sun, G. Wang, J. Li, X. Li, K. Zhang, TADF-type organic afterglow. *Angew. Chem. Int. Ed. Engl.* **60**, 17138–17147 (2021).

57. F. B. Dias, T. J. Penfold, A. P. Monkman, Photophysics of thermally activated delayed fluorescence molecules. *Methods Appl. Fluoresc.* **5**, 012001 (2017).

58. L. Gu, H. Shi, L. Bian, M. Gu, K. Ling, X. Wang, H. Ma, S. Cai, W. Ning, L. Fu, H. Wang, S. Wang, Y. Gao, W. Yao, F. Huo, Y. Tao, Z. An, X. Liu, W. Huang, Colour-tunable ultra-long organic phosphorescence of a single-component molecular crystal. *Nat. Photon.* **13**, 406–411 (2019).

59. Y. Xie, Y. Ge, Q. Peng, C. Li, Q. Li, Z. Li, How the molecular packing affects the room temperature phosphorescence in pure organic compounds: Ingenious molecular design, detailed crystal analysis, and rational theoretical calculations. *Adv. Mater.* **29**, 1606829 (2017).

60. H. Tetsuka, A. Nagoya, T. Fukusumi, T. Matsui, Molecularly designed, nitrogen-functionalized graphene quantum dots for optoelectronic devices. *Adv. Mater.* **28**, 4632–4638 (2016).

61. X. Miao, D. Qu, D. Yang, B. Nie, Y. Zhao, H. Fan, Z. Sun, Synthesis of carbon dots with multiple color emission by controlled graphitization and surface functionalization. *Adv. Mater.* **30**, 1704740 (2018).

62. N. J. Turro, V. Ramamurthy, J. C. Scaiano, *Modern Molecular Photochemistry of Organic Molecules* (University Science Books Sausalito, 2010), vol. 188.

63. J. Tan, R. Zou, J. Zhang, W. Li, L. Zhang, D. Yue, Large-scale synthesis of N-doped carbon quantum dots and their phosphorescence properties in a polyurethane matrix. *Nanoscale* **8**, 4742–4747 (2016).

64. S. Kuno, H. Akeno, H. Ohtani, H. Yuasa, Visible room-temperature phosphorescence of pure organic crystals via a radical-ion-pair mechanism. *Phys. Chem. Chem. Phys.* **17**, 15989–15995 (2015).

65. H.-B. Kim, J.-J. Kim, A simple method to measure intermolecular charge-transfer absorption of organic films. *Org. Electron.* **62**, 511–515 (2018).

66. C. Zhou, S. Zhang, Y. Gao, H. Liu, T. Shan, X. Liang, B. Yang, Y. Ma, Ternary emission of fluorescence and dual phosphorescence at room temperature: A single-molecule white light emitter based on pure organic aza-aromatic material. *Adv. Funct. Mater.* **28**, 1802407 (2018).

67. S. K. Lower, M. A. El-Sayed, The triplet state and molecular electronic processes in organic molecules. *Chem. Rev.* **66**, 199–241 (1966).

68. L. Yang, X. Wang, G. Zhang, X. Chen, G. Zhang, J. Jiang, Aggregation-induced intersystem crossing: A novel strategy for efficient molecular phosphorescence. *Nanoscale* **8**, 17422–17426 (2016).

69. M. J. Frisch, G. W. Trucks, H. B. Schlegel, G. E. Scuseria, M. A. Robb, J. R. Cheeseman, G. Scalmani, V. Barone, G. A. Petersson, H. Nakatsuji, X. Li, M. Caricato, A. V. Marenich, J. Bloino, B. G. Janesko, R. Gomperts, B. Mennucci, H. P. Hratchian, J. V. Ortiz, A. F. Izmaylov, J. L. Sonnenberg, Williams, F. Ding, F. Lipparini, F. Egidi, J. Goings, B. Peng, A. Petrone, T. Henderson, D. Ranasinghe, V. G. Zakrzewski, J. Gao, N. Rega, G. Zheng, W. Liang, M. Hada, M. Ehara, K.

Toyota, R. Fukuda, J. Hasegawa, M. Ishida, T. Nakajima, Y. Honda, O. Kitao, H. Nakai, T. Vreven, K. Throssell, J. A. Montgomery Jr., J. E. Peralta, F. Ogliaro, M. J. Bearpark, J. J. Heyd, E. N. Brothers, K. N. Kudin, V. N. Staroverov, T. A. Keith, R. Kobayashi, J. Normand, K. Raghavachari, A. P. Rendell, J. C. Burant, S. S. Iyengar, J. Tomasi, M. Cossi, J. M. Millam, M. Klene, C. Adamo, R. Cammi, J. W. Ochterski, R. L. Martin, K. Morokuma, O. Farkas, J. B. Foresman, D. J. Fox. (Wallingford, 2016).

70. M. Ohnuma, K. Hono, E. Abe, H. Onodera, S. Mitani, H. Fujimori, Microstructure of Co–Al–O granular thin films. *J. Appl. Phys.* **82**, 5646–5652 (1997).

71. H. Tetsuka, A. Nagoya, R. Asahi, Highly luminescent flexible amino-functionalized graphene quantum dots@cellulose nanofiber-clay hybrids for white-light emitting diodes. *J. Mater. Chem. C* **3**, 3536–3541 (2015).

72. S. Javanbakht, H. Namazi, Solid state photoluminescence thermoplastic starch film containing graphene quantum dots. *Carbohydr. Polym.* **176**, 220–226 (2017).

73. D. Ozyurt, S. Shafqat, T. T. Pakkanen, R. K. Hocking, A. Mouritz, B. Fox, Aggregation induced emission transformation of liquid and solid-state N-doped graphene quantum dots. *Carbon* **175**, 576–584 (2021).

74. D. Pan, L. Wang, Z. Li, B. Geng, C. Zhang, J. Zhan, L. Yin, L. Wang, Synthesis of graphene quantum dot/metal–organic framework nanocomposites as yellow phosphors for white light-emitting diodes. *New J. Chem.* **42**, 5083–5089 (2018).

75. K. Jiang, Y. Wang, X. Gao, C. Cai, H. Lin, Facile, quick, and gram-scale synthesis of ultralong-lifetime room-temperature-phosphorescent carbon dots by microwave irradiation. *Angew. Chem. Int. Ed.* **57**, 6216–6220 (2018).

76. J. He, Y. He, Y. Chen, B. Lei, J. Zhuang, Y. Xiao, Y. Liang, M. Zheng, H. Zhang, Y. Liu, Solid-state carbon dots with red fluorescence and efficient construction of dual-fluorescence morphologies. *Small* **13**, 1700075 (2017).

77. X. Sun, J. Zhao, X. Wang, W. Pan, G. Yu, J. Wang, The phosphorescence property of carbon dots presenting as powder, embedded in filter paper and dispersed in solid solution. *JOL* **218**, 116851 (2020).

78. J. Liu, N. Wang, Y. Yu, Y. Yan, H. Zhang, J. Li, J. Yu, Carbon dots in zeolites: A new class of thermally activated delayed fluorescence materials with ultralong lifetimes. *Sci. Adv.* **3**, e1603171 (2017).

79. Y. Ma, X. Zhang, J. Bai, K. Huang, L. Ren, Facile, controllable tune of blue shift or red shift of the fluorescence emission of solid-state carbon dots. *Chem. Eng. J.* **374**, 787–792 (2019).
